# *Drosophila Fatty Acid Transport Protein* Regulates Rhodopsin-1 Metabolism and Is Required for Photoreceptor Neuron Survival

**DOI:** 10.1371/journal.pgen.1002833

**Published:** 2012-07-26

**Authors:** Pierre Dourlen, Benjamin Bertin, Gilles Chatelain, Marion Robin, Francesco Napoletano, Michel J. Roux, Bertrand Mollereau

**Affiliations:** 1 Laboratory of Molecular Biology of the Cell, UMR5239 CNRS/Ecole Normale Supérieure de Lyon, UMS 344 Biosciences Lyon Gerland, Université de Lyon, Lyon, France; 2 Translational Medicine and Neurogenetics, IGBMC, UMR7104 CNRS/Université de Strasbourg/Inserm U964, Strasbourg, France; Stanford University School of Medicine, United States of America

## Abstract

Tight regulation of the visual response is essential for photoreceptor function and survival. Visual response dysregulation often leads to photoreceptor cell degeneration, but the causes of such cell death are not well understood. In this study, we investigated a *fatty acid transport protein* (*fatp*) null mutation that caused adult-onset and progressive photoreceptor cell death. Consistent with *fatp* having a role in the retina, we showed that *fatp* is expressed in adult photoreceptors and accessory cells and that its re-expression in photoreceptors rescued photoreceptor viability in *fatp* mutants. The visual response in young *fatp*-mutant flies was abnormal with elevated electroretinogram amplitudes associated with high levels of Rhodopsin-1 (Rh1). Reducing Rh1 levels in *rh1* mutants or depriving flies of vitamin A rescued photoreceptor cell death in *fatp* mutant flies. Our results indicate that *fatp* promotes photoreceptor survival by regulating Rh1 abundance.

## Author Summary

Normal vision requires precise regulation of the visual response. The deregulation of the visual response can lead to retinal diseases and blindness. The most frequent retinal disease is *retinitis pigmentosa*, and in 30%–40% of such cases rhodopsin, the light-sensitive protein, is mutated. Approximately 100 rhodopsin mutations have been identified; they affect folding, trafficking, and activity of the rhodopsin protein and induce consequent photoreceptor neuron death. Despite extensive studies, many aspects of retinal degeneration remain unclear. *Drosophila* is a suitable model organism to study retinal diseases. To understand the mechanisms of retinal degeneration, we studied the previously uncharacterized *fatty acid transport protein* (*fatp*) gene in *Drosophila*. We found that flies that lack the *fatp* gene exhibit a deregulation of the visual response and an adult-onset and progressive retinal degeneration. In addition, we show that such retinal degeneration is due to the death of photoreceptor neurons in which rhodopsin proteins accumulate. In summary, we report a novel fly model of adult-onset retinal degeneration. We uncovered a novel and interesting mechanism by which Fatp, a potential regulator of lipid transport and metabolism, is responsible for the regulation of rhodopsin levels in the photoreceptor neurons.

## Introduction

Retinal degeneration is a major health concern that affects one in 2000 people worldwide (http://www.sph.uth.tmc.edu/Retnet/) [1]. Although human retinopathies are heterogeneous in physiopathology and severity, they all involve loss of photoreceptor (PR) neurons, which leads to blindness. The most frequent retinal disease is retinitis pigmentosa, which is caused by mutations in one or more of at least 54 distinct genes (Retnet). Among them, the most frequently mutated gene is *rhodopsin* (*rho* in mammals) which is mutated in 30–40% of all cases of autosomal dominant retinitis pigmentosa (ADRP) (Retnet) [2]. Rhodopsin is the light-sensitive protein of PRs that activates phototransduction. Approximately 100 *rho* mutations have been identified, and they affect folding, trafficking and activity of the rhodopsin protein. Despite extensive study, the mechanisms of retinal degeneration remain unclear.

Retinal degeneration has been studied extensively in *Drosophila* [3], [4], [5]. Many mutations in *Drosophila* phototransduction pathway genes induce PR degeneration. The mechanism of toxicity of these mutations is related either to a defect in folding, trafficking or activity of the *Drosophila* Rhodopsin-1 protein (Rh1), or to an accumulation of toxic Rh1-Arrestin2 (Arr2) complexes, or to a deregulation of the Ca^2+^ homeostasis [4], [5]. In addition, mutations can be introduced in *Drosophila* genes to model human diseases. For example, the *rh1^P37H^* allele, which corresponds to *rho^P23H^*, the most frequent mutation of *rhodopsin* in ADRP, has been successfully introduced into *Drosophila* and induces PR degeneration [6]. We recently used *Drosophila* to identify genes involved in PR survival [7]. Using a new method of PR visualization, called Tomato/GFP-FLP/FRT, we screened recessive lethal mutations and found *fatp^k10307^* to be associated with PR cell death. The *fatp^k10307^* mutation consists of the insertion of a P{lacW} element into the open reading frame of the *fatty acid transport protein* (*fatp*) gene, which has never been characterized in *Drosophila*. Its closest mammalian orthologs are *fatp1* and *fatp4* [8]. The mammalian members of the Fatp family of proteins have acyl-CoA synthetase enzymatic activity and facilitate cellular fatty acid uptake [9], [10]. Each member of the family has a specific expression pattern and function. *fatp1* is expressed in muscle, heart, brain, adipose tissue and retina [11], [12], and is involved in thermogenesis and obesity. *fatp4* is expressed most abundantly in the small intestine, brain, skeletal muscle, heart, skin, liver and kidney [10], [13], [14]. Loss of *fatp4* in mice or in humans is associated with restrictive dermopathy related to the ichthyosis prematurity syndrome, a skin defect caused by altered lipid and fatty acid compositions [15], [16], [17].

In this work, we show that *Drosophila fatp* is required for PR survival. PR degeneration in a *fatp* null mutant is adult-onset and progressive. The onset of degeneration in the *fatp* mutant correlates with the time of expression of *fatp* in the normal adult retina. We then investigated the mechanisms of PR degeneration in the *fatp* mutant. We show that PRs in the *fatp* mutant exhibit an elevated photoresponse that is associated with high levels of Rh1. We also found that *fatp* mutant flies have a defect in Rh1 degradation and that reducing the level of Rh1 restored PR survival.

## Results

### *fatp* is required for PR viability in adult *Drosophila*

**Figure 1:**
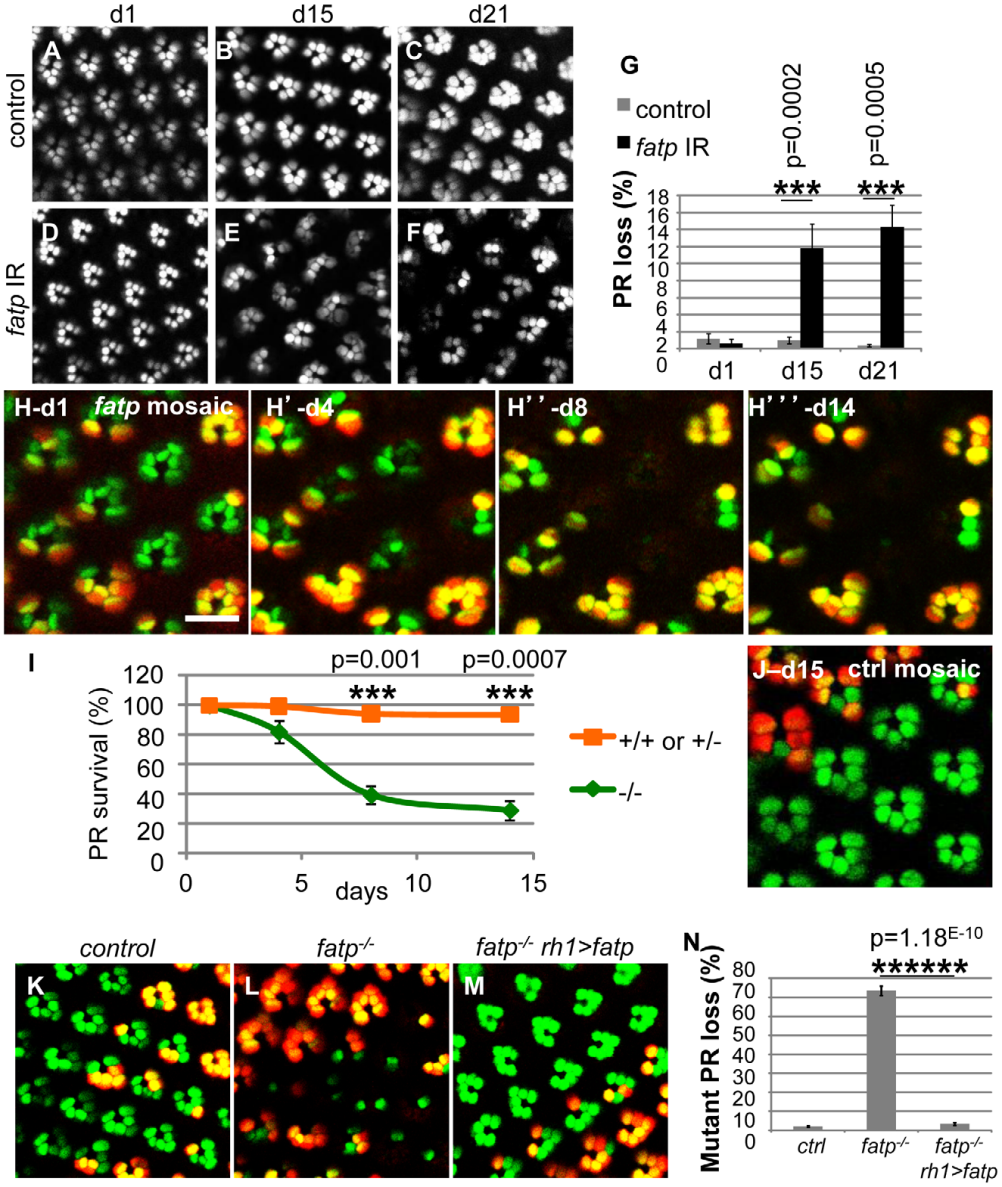
Loss of *fatp* induces adult-onset progressive degeneration of PRs. (A–G) Consequences of *fatp* knockdown by RNA interference on PR viability. (A, B, C) Visualization of PRs using a cornea-neutralization method for 1-, 15- and 21-day-old PRs expressing *lacZ* as a control. (D, E, F) PRs expressing the *fatp*-interfering RNA and (G) PR quantification. Whereas all PRs were present in control retina and *fatp*-knocked-down retina at day one (A, D), there were significant PR losses at days 15 and 21 in *fatp*-knocked-down retina (B vs E, C vs F, t-test, n≥6). (H) Time-course analysis of *fatp^k10307^* mutant mosaic retina using the Tomato/GFP-FLP/FRT method and (I) quantification. All outer PRs (R1–R6) expressed GFP (green). FLP-mediated mitotic mutant clones were visualized by the absence of rh1-tdTomato (red). Clones of the same eye were visualized in the same fly at day one, four, eight and fourteen after hatching. (H) All PRs were present at day one (scale bar = 10 µm). (H′) From day four, mutant PRs (in green) started to disappear. (H″ and H′″) Losses of mutant PRs were significant at day eight and day fourteen (paired t-test, n = 3). (I) Quantified results are expressed as mean ± SD. (J) Analysis of a control 15-day-old mosaic retina using the Tomato/GFP-FLP/FRT method. All PRs are present. (K–N) Rescue of *fatp^k10307^*-mutant PRs by re-expression of wild-type *fatp*. (K) Tomato/GFP-FLP/FRT visualization of 10- to 14-day-old control, (L) *fatp^k10307/k10307^* and (M) *fatp^k10307/k10307^+rh1>fatp* mosaic retinas and (N) quantification (t-test, n> = 5). In the control (K), all PRs were present. In *fatp* mutant mosaic retina (L), 70% of the mutant PRs were lost. In these retinas, mutant PRs were rescued by re-expression of *fatp* under the control of the *rh1* promoter (M).

To study the role of *fatp* in PR viability, we first used RNA interference to reduce *fatp* expression. *fatp*-interfering RNA was specifically produced in the retina under the control of eye-specific drivers (ey-Gal4 and GMR-Gal4) (Figure 1A–1G). *fatp* knockdown led to a progressive loss of PRs, indicating that *fatp* expression is required for PR viability. To validate these findings, we used the *fatp^k10307^* mutation, generated by the insertion of a 10.7 kb P{lacW} element into the first exon of the gene. This mutation is recessive and can be considered a null allele as it completely prevents *fatp* expression, abolishing the production of *fatp* mRNA and protein in homozygous *fatp^k10307^* L1 larvae (Figure S1). As a consequence, *fatp^k10307^* mutant larvae died during the second instar. To study the role of *fatp* in adult PR and circumvent *fatp^k10307^* larval lethality, we used the Tomato/GFP-FLP/FRT mosaic method [7]. This new method combines mitotic recombination and cornea neutralization techniques [18], [19], [20] to allow time-course analysis of homozygous mutant clones in living flies over several days. We evaluated PR presence, identified on the basis of GFP expression, for 14 days from hatching in *fatp^k10307^* mutant retinas (Figure 1H–1J). All *fatp* mutant PRs were present upon hatching but progressively disappeared starting on day four (Figure 1H, 1H′). Losses of *fatp* mutant PRs were statistically significant in the retinas of 8- and 14-day-old adults (Figure 1H″, 1H′″, 1I). In contrast, little or no PR loss occurred in neighboring wild-type or heterozygous tissue, indicating that the *fatp^k10307^* mutation was cell-autonomous (Figure 1H, 1I, 1J).

The cell-autonomous nature of the PR loss in the *fatp* mutant (Figure 1H–1I) suggested that *fatp* expression is required for PR viability. To confirm this requirement, we attempted to rescue PR viability in the *fatp^k10307^* mutant with tissue-specific expression of wild-type *fatp* using the UAS/GAL4 system [21]. *fatp* re-expression from the *rh1* promoter in the R1–R6 PRs fully rescued the *fatp^k10307^* mutant PR phenotype (Figure 1K–1N). These results demonstrate that *fatp* expression in PRs is required for their survival.

**Figure 2:**
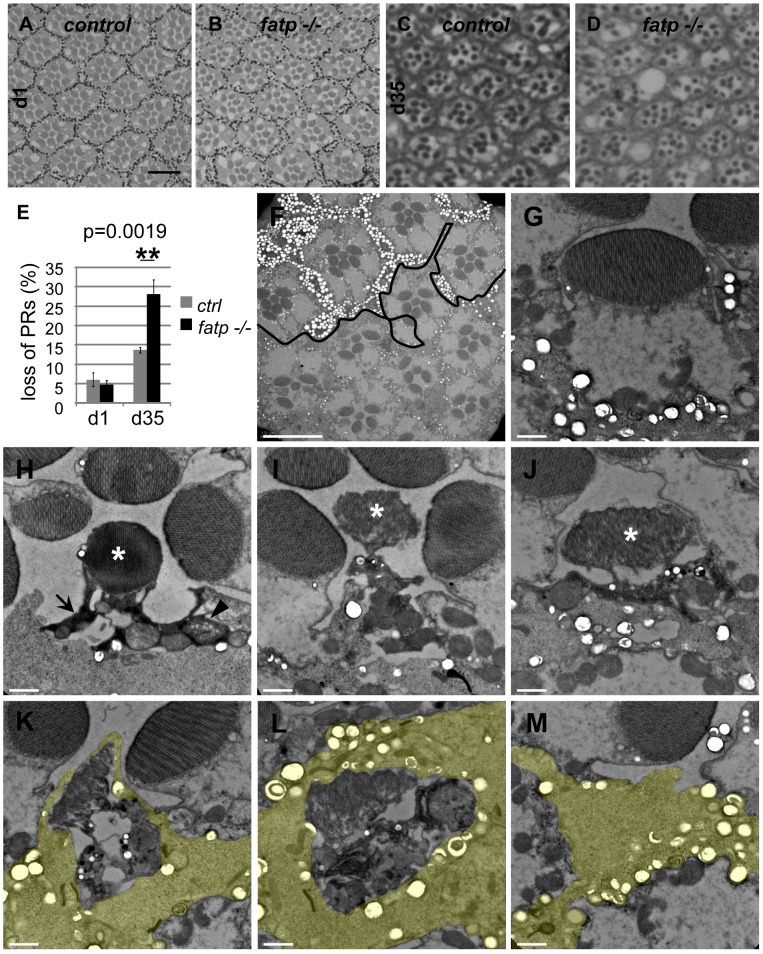
Histological analysis of *fatp^−/−^* PR degeneration. (A–E) Analysis of *fatp^k10307^* mutant PR survival using resin-embedded tangential sections. (A, C) Control retina of 1- and 35-day-old flies (scale bar = 10 µm). (B, D) Homozygous *fatp^k10307^* retina of 1-day-old and 35-day-old flies. (E) PR losses in *fatp* mutant retinas were significant at 35 days compared to those in control retinas (t-test, n = 6). (F–M) Electron microscopy analysis of *fatp^−/−^* PR degeneration. *fatp^k10307^* mosaic retina of 15-day-old flies were analyzed. (F) Wild-type and heterozygous clones were marked with numerous large pigment granules in the IOCs and at the basis of PR rhabdomeres (white spots, above the black line) whereas homozygous mutant part exhibited rare small pigment granules (under the black line, scale bar = 10 µm). Several PRs were missing in the homozygous mutant part. (G–M) Different stages of *fatp^k10307/k10307^* PR degeneration (scale bar = 1 µm). (G) *fatp^k10307/k10307^* PR exhibiting no sign of degeneration. (H) The cytoplasm of *fatp^k10307/k10307^* PRs shrank and became electron-dense (arrow). Some mitochondria were swelling (arrowhead). The rhabdomere was not much affected (*). (I–J) PRs then disintegrated. This was clearly visible at the level of the rhabdomere (*). (K, L, M) Degenerating PRs were finally phagocytosed and digested by the neighboring interommatidial cell (yellow).

Next, we performed structural and ultra-structural analyses of *fatp* mutant PR in resin-embedded retina sections. We used classical brightfield microscopy to examine PR integrity in whole-eye *fatp* mutant clones generated by the *EGUF/Hid* method [22] (Figure 2A–2E). PR loss started after approximately 15 days and increased progressively with age (Figure 2A–2E). Using this approach, PR loss was detected later than with the Tomato/GFP-FLP/FRT method. This may be because the Tomato/GFP-FLP/FRT method is based on the expression and targeting of GFP in the rhabdomere and thus detects early deficits whereas classical histological methods assess only the physical presence or absence of PRs. We also detected progressive and adult-onset PR loss by recording the number of nuclei between the apical and basal layers of *fatp* mutant retinas in horizontal cryosections (Figure S2). We then studied *fatp* mosaic retinas by electron microscopy (EM) to characterize further PR degeneration in *fatp* mutant cells (Figure 2F–2M). On EM images, we could distinguish homozygous *fatp* mutant PRs from the absence of pigment vesicles at the base of the rhabdomeres and in inter-ommatidial cells (IOCs) (Figure 2F). We observed several levels of PR degeneration among *fatp* mutant cells ranging from normal PRs to PRs that were fully degenerated and engulfed by neighboring IOCs. Most normal PRs displayed no obvious sign that would predict future degeneration (Figure 2G). Some PRs were electron-dense and their cytoplasm was contracted suggesting that they had initiated the degeneration process (Figure 2H). In these PRs, some swelling mitochondria could be observed but the rhabdomeres were still intact. In more advanced stages of degeneration, the rhabdomeres were clearly affected with disorganized microvilli structures (Figure 2I, 2J). In addition, the neighboring IOCs seemed to be activated with a typical spotted pattern in the cytoplasm. At the end of the degenerative process, PRs were phagocytosed by the neighboring IOCs and apparently digested (Figure 2K–2M). Thus, the absence of *fatp* causes progressive adult-onset PR degeneration.

### *fatp* is expressed in the adult retina

**Figure 3:**
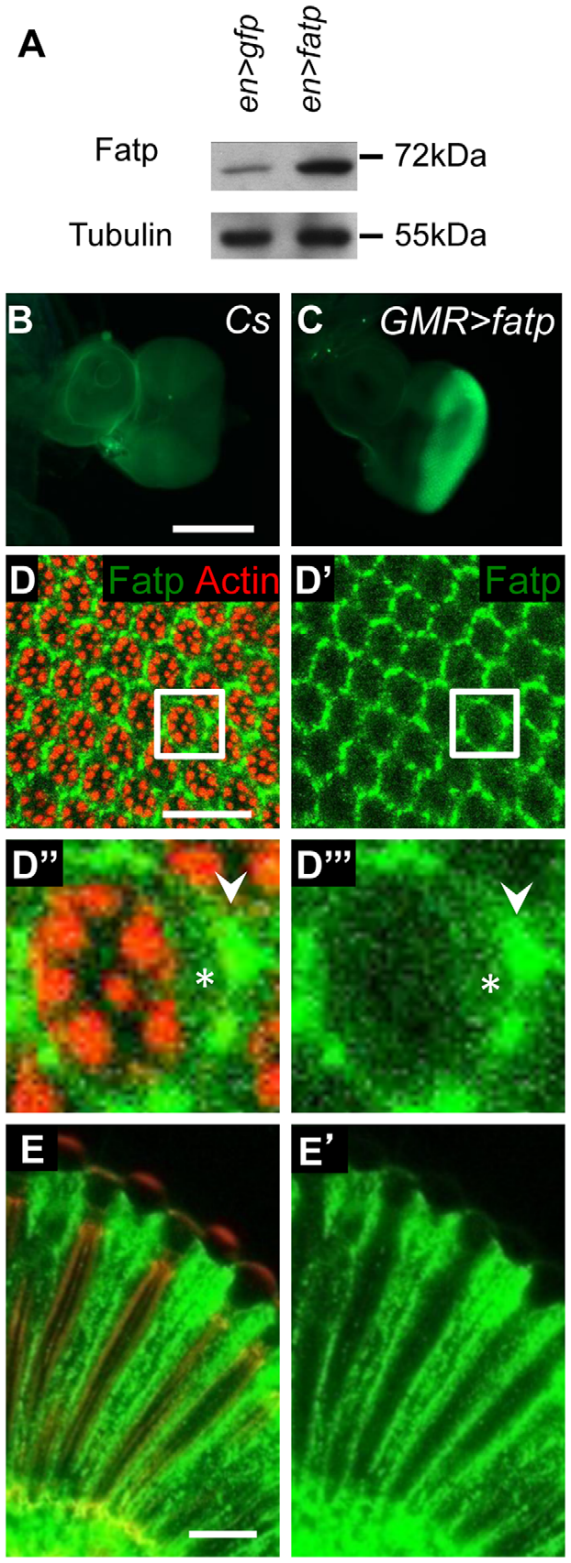
*fatp* is expressed in the adult retina. (A) Western blot analysis of en-GAL4 UAS-GFP (en>GFP) and en-GAL4 UAS-fatp (en>fatp) adult eye extracts using anti-Fatp C11-7 and anti-tubulin antibodies. Anti-Fatp C11-7 detects a single band at 72 kDa, which corresponds to the predicted molecular weight of Fatp. Endogenous and ectopically expressed Fatp were detected. Tubulin was used as a loading control. (B, C) Immunofluorescent detection of Fatp in wild-type (CS) and GMR-GAL4 UAS-fatp (GMR>fatp) third instar larva eye imaginal disc using the anti-Fatp C11-7 antibody (scale bar = 200 µm). (C) Fatp was detected in the posterior part of the disc, which corresponds to the expression profile of the GMR promoter. (D–E) Immunostaining of endogenous Fatp and Actin in whole-mount *cnbw* adult retina using the anti-Fatp C11-7 antibody and phalloidin. In the tangential (D) and longitudinal plans (E), Fatp immunostaining is detected in the cytoplasm of PRs (star) and accessory cells (arrowhead). Rhabdomeres are stained with phalloidin (scale bar = 20 µm).

We determined the specificity of *fatp* expression in developing and adult *Drosophila* eyes. To do this, we generated an anti-Fatp antibody (C11-7) that was sensitive enough to detect ectopic Fatp and endogenous Fatp on western blots and in immunofluorescence experiments (Figure 3A–3E). No *fatp* expression was observed in third instar eye imaginal discs (Figure 3B), but we detected Fatp protein in PR cytoplasm juxtaposed to PR rhabdomeres and in IOCs of the adult retina (Figure 3D, 3E). To capture the *fatp* expression profile, we exploited the *fatp^k10307^* enhancer trap line, which carries the β-galactosidase reporter (Figure S3). Using a β-galactosidase antibody, we detected β-galactosidase in the nuclei cell layer that includes the outer PRs and accessory cells in adult head cryosections (Figure S3A–S3D). Immunolocalization experiments revealed that small amounts of Fatp consistently co-localized with the neuronal marker ELAV in the outer PRs and that Fatp was also present in apical IOCs (Figure S3A–S3D). Thus, the *fatp^k10307^* enhancer trap line faithfully reproduces the distribution of Fatp and can be used to follow *fatp* expression. Testing for β-galactosidase activity in this line revealed that *fatp* was also expressed in the adult optic lobe around the medulla and lamina, in the midgut and in the salivary glands but not in the eye imaginal disc of third instar larvae (Figure S3E–S3L). In conclusion, the absence of *fatp* expression in the developing eye disc and its presence in differentiated PRs are consistent with *fatp* being required for the viability of PRs in adult flies.

### *fatp* regulates the visual response

**Figure 4:**
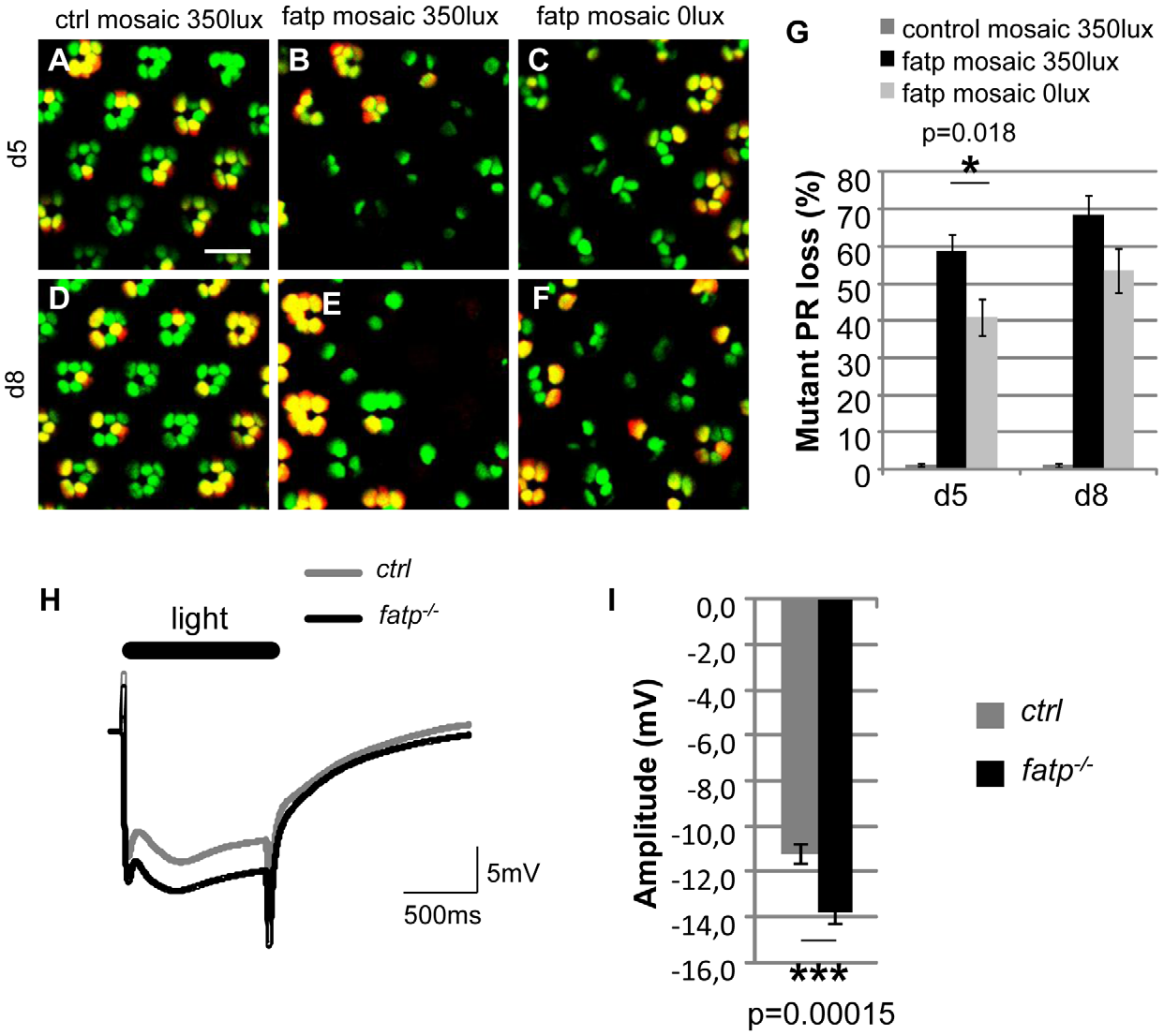
The visual response is altered in *fatp* mutant PRs. (A–F) Tomato/GFP-FLP/FRT visualization of *fatp^k10307^* mutant mosaic retinas from 5- and 8-day-old flies reared from the pupal stage in normal room light (350 lux) or in darkness (0 lux) (scale bar = 10 µm). (G) Quantification of *fatp^k10307^* mutant PR loss. (A, D) No PR losses occurred in control mosaic retinas from 5-day-old and 8-day-old flies reared in room light conditions. (B, E, G) In *fatp* mutant retinas from light-reared flies, 58.8±4.5% and 68.5±4.9% of the mutant PRs were lost in 5-day-old and 8-day-old flies, respectively. (C, F, G) PR losses were significantly lower in flies reared in the dark (t-test, n = 8). (H) ERG recordings from control and *fatp^k10307/k10307^* 8-day-old retinas and (I) quantification. Retinas were exposed to a one second flash of orange light. (I) The amplitude of the plateau is significantly higher in the *fatp* mutant retina than in the control retina (t-test, n = 24).

We investigated whether the requirement for *fatp* expression for PR viability is related to the visual response. First, we tested whether PR degeneration in the *fatp* mutant is light-dependent. *fatp* mutant flies reared in normal light conditions exhibited significantly greater PR losses than *fatp* mutant flies reared in complete darkness (Figure 4A–4G). This indicates that light contributes to PRs loss in the *fatp* mutant. Then, to examine directly the visual response in the *fatp* mutant, we performed electoretinogram (ERG) recordings on white-eyed flies (Figure 4H, 4I): we used 8-day-old flies, an age at which the integrity of *fatp* mutant PRs is still intact as observed in resin-embedded retinal sections (data not shown). We obtained ERG recordings that measure the summed responses of all retinal cells. Wild-type flies exhibited a corneal negative receptor potential in response to orange light, which returned to baseline when the light was switched off (Figure 4H). The amplitude of the ERG was higher in *fatp^k10307^* flies than controls, with no apparent difference in the kinetics of the visual response (Figure 4H, 4I). These results suggest that Fatp is a negative regulator of the visual response.

### The elevated levels of Rh1 cause PR loss in the *fatp* mutant

**Figure 5:**
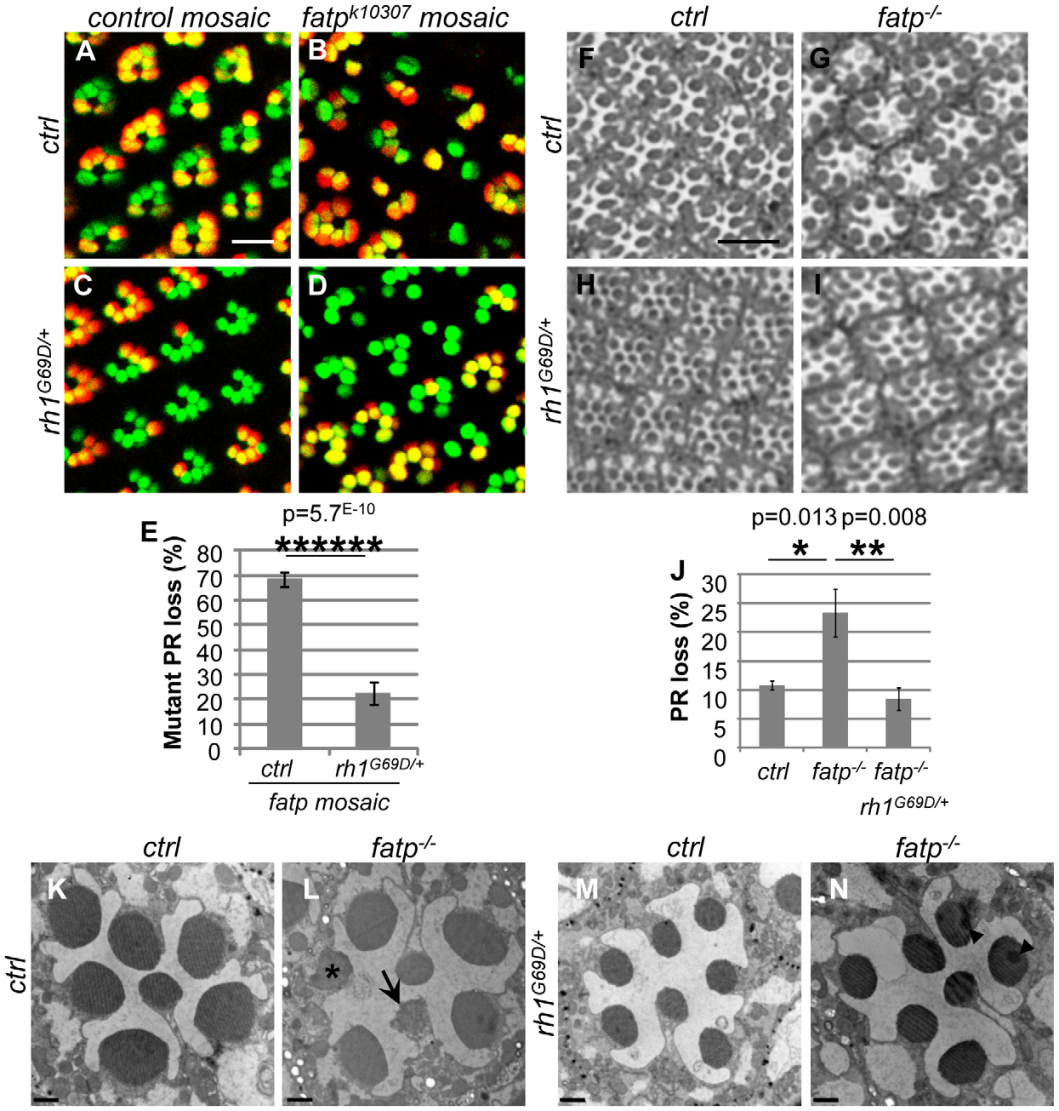
*Rh1^G69D^* rescues PR viability in the *fatp* mutant. (A–E) Analysis of the survival of control, *fatp^k10307^*, *rh1^G69D/+^* and *fatp^k10307^rh1^G69D/+^* double mutant PRs using the Tomato/GFP-FLP/FRT method in 15-day-old flies (scale bar = 10 µm). (E) Quantification of mutant PR losses (t-test, n≥12). PR loss in the *fatp^k10307^ rh1^G69D/+^* double mutant was dramatically lower than in the *fatp* single mutant. (F–J) Analysis of the survival of control, *fatp^k10307^*, *rh1^G69D/+^* and *fatp^k10307^ rh1^G69D/+^* double mutant PRs using resin-embedded tangential sections in 28-day-old flies (scale bar = 10 µm). (J) Quantification of PR loss. Significantly more PRs died in the *fatp* mutant than in control retina (t-test, n = 6). In the double mutant, PR losses were reduced to control levels. (K–N) Electron microscopy analysis of whole-eye control (K), *fatp^k10307^* (L), *rh1^G69D/+^* (M) and *fatp^k10307^ rh1^G69D/+^* (N) mutants in 28-day-old flies (scale bar = 1 µm). Whereas PRs degenerated in *fatp^k10307^* ommatidia (arrow) and were phagocytosed by IOCs (*), PRs survived in *fatp^k10307^ rh1^G69D/+^* double mutant flies. Rhabdomeres of *rh1^G69D/+^* and *fatp^k10307^ rh1^G69D/+^* outer PRs were reduced in size. Arrowheads show artifactual shadows on the sample.

We wondered whether PR degeneration in the *fatp* mutant could be rescued by *rh1* mutant alleles as previously described for light-dependent retinal degeneration mutants such as *rdgC* and *norpA* [23]. We therefore tested whether PR viability in the *fatp* mutant could be rescued by *rh1^G69D^* and *rh1^I17^* (*ninaE^G69D^* and *ninaE^I17^*) alleles (Figure 5 and Figure S5). *rh1^G69D^* is a dominant negative allele whereas *rh1^I17^* is a null allele. Although the *rh1^G69D^* allele causes retinal degeneration in old flies under constant light exposure, it could be used in our study because these mutants raised under a 12 h day/light cycle did not exhibit retinal degeneration (Figure 5C, 5H, 5M; Figure S4; and [23], [24]). Using the Tomato/GFP-FLP/FRT method, we compared PR loss in retinas in the single *fatp^k10307/k10307^* mutant to that in *fatp^k10307/k10307^rh1^G69D/+^* double mutant. PR loss was very much lower in *fatp^k10307/k10307^rh1^G69D/+^* mutant retinas than in *fatp^k10307/k10307^* mutant retinas (Figure 5A–5E). In resin-embedded eye sections, PR viability was found to be fully rescued in the *fatp^k10307/k10307^rh1^G69D/+^* double mutant (Figure 5F–5J). EM showed that despite the smaller size of the rhabdomeres, PR ultra-structure was fully rescued in *fatp^k10307/k10307^rh1^G69D/+^* double mutants (Figure 5K–5N). Similarly, a rescue of PR degeneration was observed in *fatp^k10307/k10307^rh1^I17/+^* double mutant (Figure S5). Thus, *rh1* mutant alleles rescued PR degeneration in the *fatp* mutant.

**Figure 6:**
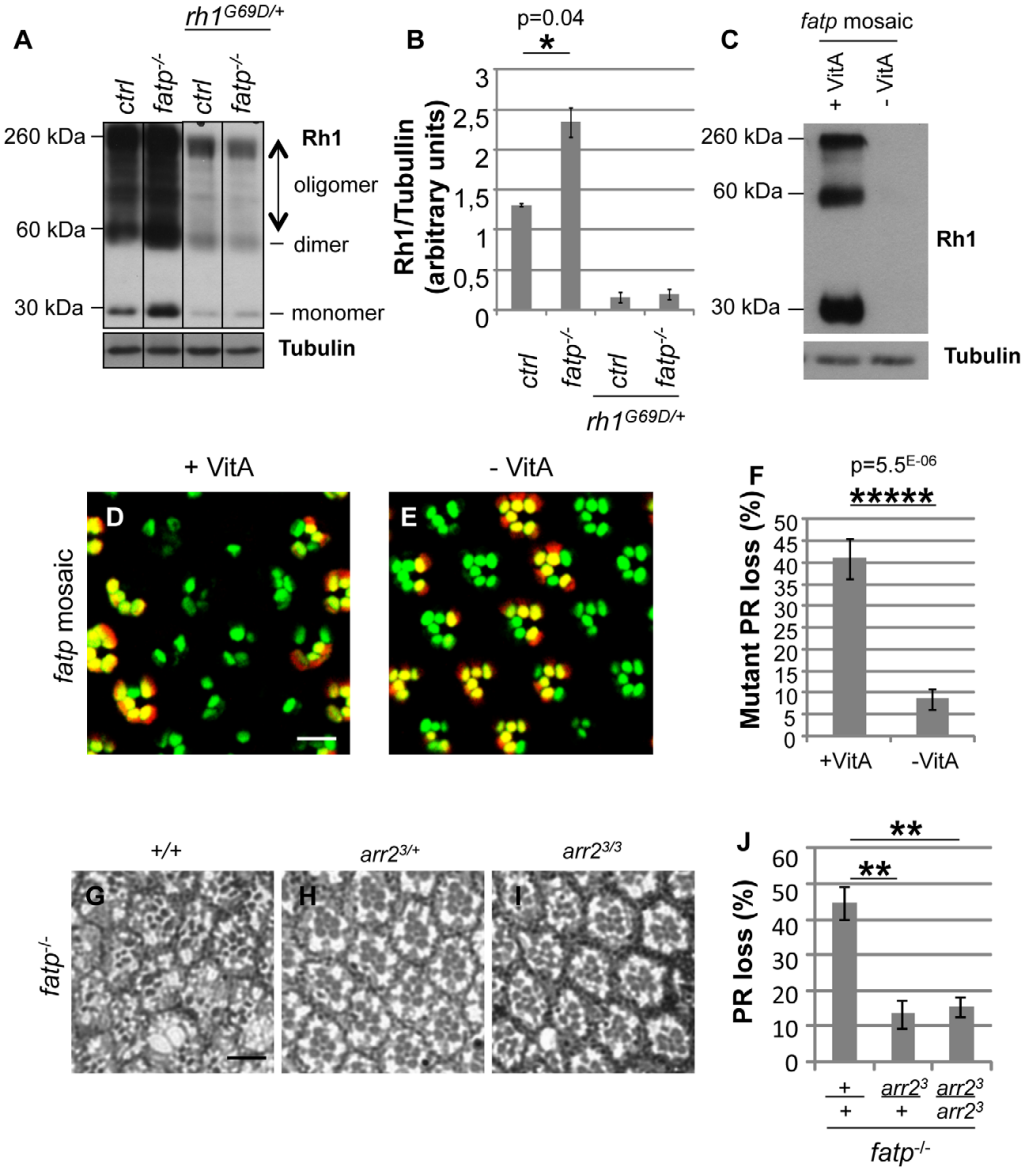
Elevated Rh1 levels are responsible for PR loss in the *fatp* mutant. (A) Western blot analysis of Rh1 in boiled head extracts from control, *fatp^k10307/k10307^*, *rh1^G69D/+^* and *fatp^k10307/k10307^*/*rh1^G69D/+^* 1 to 11-day-old flies. Tubulin was used as a loading control. (B) Quantification of protein levels. Dimer and oligomer forms of Rh1 were due to the boiling of the extracts. Rh1 levels were twofold higher in the *fatp* mutant than in the control. The level of Rh1 was substantially lower in the *rh1^G69D/+^* single mutant and in the *fatp^k10307/k10307^*/*rh1^G69D/+^* double mutant. (C) Western blot analysis of Rh1 and tubulin in *fatp* mutant mosaic retina extracts from 5-day-old flies reared from the embryonic stage on control (+VitA) and vitamin A-deficient (-VitA) media. Tubulin was used as a loading control. Rh1 was not detectable in flies reared on vitamin A-deficient medium. (D, E) Visualization of *fatp* mutant mosaic retina of 5-day-old flies reared from the embryonic stage on control (D) and vitamin A-deficient (E) media using the Tomato/GFP-FLP/FRT method (scale bar = 10 µm). (F) Quantification of mutant PR loss (t-test, n≥12). *fatp* mutant PR viability was dramatically restored in flies reared on vitamin A-deficient medium. (G–J) Analysis of the loss of *fatp^k10307^*, *fatp^k10307^ arr2^3/+^* and *fatp^k10307^ arr2^3/3^* double mutant PRs using resin-embedded tangential sections in 34-day-old flies (scale bar = 10 µm). (H) Quantification of PR loss. In the double mutants retinas, PRs were significantly rescued (p = 0.0021 and p = 0.0016, t-test, n = 6).

We tested whether *rh1* is dysregulated in *fatp* mutant PRs. We assayed Rh1 protein in *fatp* mutant and control retinas by western blotting (Figure 6A, 6B). The Rh1 content of *fatp^k10307/k10307^* mutant retinas was double that of control retinas. We confirmed that Rh1 was less abundant in *fatp^k10307/k10307^rh1^G69D/+^* double mutant than *fatp^k10307/k10307^* single mutant retinas (Figure 6A, 6B). These results indicate that Rh1 metabolism is altered in the *fatp* mutant and that reducing Rh1 levels in *fatp* mutants protects the PRs. To confirm this conclusion, we examined whether rearing flies on a vitamin A-deficient medium could reduce PR loss in *fatp* mutant retinas (Figure 6C–6F). Vitamin A is the precursor of the Rh1 chromophore and is required for Rh1 synthesis. A vitamin A-deficient diet rescues retinal degeneration in several mutants, including *rdgB*, *crumbs*, and *arrestin* mutants [25], [26], [27]. In retinas of flies reared on a vitamin A-deficient diet, occasional PR degeneration was visible but most rhabdomeres were present (Figure S6). *fatp* mutant flies deprived of vitamin A produced no detectable Rh1 protein and the PR loss in these flies was very much lower than that in flies receiving a standard diet (Figure 6C–6F). Thus, reducing the Rh1 level rescued PR degeneration in the *fatp* mutant. Therefore, PR degeneration in the *fatp* mutant appears to be due to an over abundance of Rh1.

To test whether high levels of Rh1 are toxic to the PR, we overexpressed *rh1* in developing and adult PRs (Figure S7). Ectopic expression of *rh1* with the GMR driver led to the rough eye phenotype, indicating that rh1 overexpression is toxic in the developping eye (Figure S7A, S7B). We also overexpressed *rh1* specifically in differentiated R1-6 PRs using the *rh1* promoter. *rh1* overexpression led to rhabdomere degeneration (Figure S7C, S7D). Thus, we conclude that elevated Rh1 levels in *fatp* mutant PRs can lead to PR pathogenesis.

Next we examined the possible involvement of Arrestin2 (Arr2) in Rh1 toxicity in *fatp* mutants. Stable Rh1-Arr2 complexes are toxic in adult PRs and are responsible for retinal degeneration in several mutants including *norpA*, *rdgC*, *rdgB* [26], [28], [29], [30]. To examine this possibility, we tested for genetic interaction between *fatp^k10307^* and *arr2^3^* mutations (Figure 6G–6J). We found that PRs were rescued in *fatp* and *arr2* double mutant retina, indicating that *arr2* is required for the toxicity in *fatp* mutant PRs. Together with the excess of Rh1 detected in *fatp* mutant, these observations suggest that toxic Rh1-Arr2 complexes induce PR degeneration in the *fatp* mutant.

We tested whether the elevated Rh1 levels in *fatp* mutant PRs were due to deregulation of *rh1* expression and/or degradation of Rh1. We first analyzed *rh1* expression in *fatp* mutant retinas using a *rh1-lacZ* reporter to monitor *rh1* transcription. We did not detect higher levels of lacZ in *fatp* mutant retinas than in control retinas (). This shows that *rh1* transcription is not upregulated by the *fatp* mutation. Presumably, therefore, *fatp* regulates Rh1 levels post-transcriptionally. To test for the involvement of *fatp* in Rh1 degradation, we forced Rh1 degradation by illuminating the retina with blue light as previously described [31]. Blue illumination maintains Rh1 in an active conformation and induces its degradation. In white-eyed control flies blue light illumination for 6 h induced a significant loss of Rh1 for the retinas whereas the same treatment of white-eyed *fatp* mutants did not result in decreased Rh1 abundance (Figure S8C, S8D). This suggests that light induced-Rh1 degradation is impaired in *fatp* mutant PRs and this may explain why there is more Rh1 in these retinas than controls.

## Discussion

Mutations resulting in inactive Rh1, impaired visual responses and PR degeneration have been studied extensively [4], [5]. In this study, we describe the phenotype of *fatp^k10307^*, the first mutation known to exhibit elevated Rh1 levels leading to loss of PRs.

We show that *fatp* expression is required for PR viability in adult *Drosophila*. In the absence of *fatp*, PRs degenerate progressively during adulthood. Moreover, we demonstrate that the requirement for *fatp* in adult PRs is cell-autonomous, which is in agreement with the presence of Fatp in adult PRs and with its absence from the developing eye imaginal disc. The age-dependent PR degeneration in the *fatp* mutant is reminiscent of *Drosophila* models of ADRP [3], [6]. We thus propose that *fatp*-associated degeneration is a new model of late-onset PR degeneration.

Our results indicate that PR death in *fatp* mutants is a consequence of elevated levels of Rh1. We demonstrate that reducing Rh1 levels, as a consequence of *rh1* mutation or a vitamin A-deficient diet, efficiently restored PR viability in *fatp* mutants (Figure 5, Figure 6, and Figure S5). Thus, the accumulation of Rh1 is toxic for the PRs in *fatp* mutants. One possibility is that Rh1 associates with Arr2, forming toxic Rh1-Arr2 complexes as in *norpA*, *rdgC*, *rdgB* mutants [26], [28], [29], [30]. Indeed, we found that disrupting either *rh1* or *arr2* rescued PR loss in *fatp* mutants (Figure 5, Figure 6, and Figure S5). This genetic evidence is consistent with Rh1-Arr2 complexes causing PR degeneration in *fatp* mutant. Definitive proof of this mechanism requires the direct assessment of Rh1/Arr2 complexes in *fatp* mutants. Also, we cannot exclude the existence of additional toxic mechanisms. For example, the elevated visual response in *fatp* mutants (Figure 4) may contribute to PR death because of a defect in Ca^2+^ homeostasis.

We found that Rh1 protein levels were elevated in *fatp* mutant retinas and that this was probably due to decreased Rh1 degradation (Figure S8C, S8D). *fatp* may regulate sphingolipid metabolism, which controls Rh1 trafficking and degradation [32], [33]. In support of this hypothesis, the total ceramide content is higher in the skin of *fatp4^−/−^* than control mice [15]. Ceramidase facilitates the endocytic turnover of Rh1 and rescues retinal degeneration in *arr2* and *phospholipase C* mutants [32], [34]. Similarly, *Drosophila* Fatp may limit ceramide levels and inhibit endocytic turnover of Rh1. It is also possible that *fatp* regulates Rh1 synthesis, but we show that loss of *fatp* does not affect *rh1* gene transcription (Figure S8A, S8B). Alternatively, Fatp may regulate the synthesis or recycling of the retinal chromophore required for Rh1 synthesis. In mammals, Fatp1 inhibits two enzymes of the visual cycle *in vitro*, LRAT and RPE65, which respectively produce and consume retinyl-ester, a fatty acid-linked form of the chromophore [12]. A similar visual cycle was recently described in *Drosophila* [35], [36], [37]. Therefore, chromophore synthesis or recycling may be increased in the *fatp* mutants, resulting in upregulated Rh1 synthesis. In support of this hypothesis, we showed that inhibiting chromophore synthesis in vitamin A-deficient medium fully rescued PR viability in *fatp* mutant retinas (Figure 6D–6F). Nevertheless, the mechanisms by which *fatp* may regulate the visual cycle remain to be elucidated.

The visual response was higher in the *fatp* mutant retina than control retina but the mechanisms involved are unclear. Previous work has shown that the ERG amplitude depends on the ratio between the level of Rh1 and the Rh1 kinase activity of *gprk1* [38]: decreasing *gprk1* activity resulted in higher ratios and elevated ERG amplitudes whereas increasing *gprk1* activity resulted in lower ratios and lower ERG amplitudes [38]. Whether there is a disequilibrium between phosphorylated/unphosphorylated forms of Rh1 in *fatp* mutant retinas remains to be explored. Alternatively, *fatp* may be required for the production of lipid metabolites that regulate the phototransduction cascade. Indeed, it has been suggested that polyunsaturated fatty acids, which are potential diacyl-glycerol metabolites, act on TRP/TRPL channels [39], [40], [41]. Thus, the elevated visual response may be the consequence of a lipid metabolite dysregulation in *fatp* mutant retinas.

In conclusion, *fatp* mutation is a new model of retinal pathology in flies in which the up-regulation of Rh1 contributes to progressive PR degeneration. Whether a similar pathological mechanism exists in human retinal diseases remains to be determined.

## Materials and Methods

### *Drosophila* stocks

For RNA interference, the *UAS-dicer; ey-Gal4*, *GMR-Gal4/Cyo; rh1-GFP* line (kind gift of C Desplan) was crossed with the *UAS-RNAi* line against *fatp* (VDRC #48719). The *FRT40A fatp^k10307^*/*Cyo* and *rh1-Gal4, ey-FLP; FRT40A rh1-tomato^ninaC^/Cyo; UAS-GFP^ninaC^* lines were described previously [7]. The *ninaE^G69D^* line (named *rh1^G69D^* in the text) was previously used in [42]. The *rh1^I17^* (*ninaE^I17^*) allele was obtained from bloomington (BL#5701). Whole-eye mutant clones were generated using the; *FRT40A GMR-hid CL EGUF/Cyo*; line [22]. The *UAS-rh1* and *arr2^3^* lines were a kind gift of HD Ryoo and N Colley respectively. To obtain white-eyed flies carrying *Pw+* transgenes, we used the *pWIZ* construct that expresses an iRNA against the white gene [43]. Flies were reared on standard corn medium at 25°C in a 12-h light/12-h dark environment unless noted otherwise. Vitamin A-deficient medium contained yeast (12 g), agar (1,5 g), sucrose (7,5 g), cholesterol (0,03 g), sodium methyl-4-hydroxybenzoate (1.15M, 3.75 mL) and propionic acid (0.72 mL) in distilled water (150 mL).

### Generation of UAS-fatp transgenic line

*fatp* cDNA (SD05207, Gold cDNAs Collection) was recovered from BDGP DGRC in pOT2 vector. *fatp* cDNA was cloned (XhoI/EcoRI) into a pUAST-w+-attB transgenic fly vector. Best Gene, Inc (CA, USA) generated transgenic lines using PhiC31 integrase-mediated transgenesis [44]. The vector DNA was injected in embryos carrying attP docking sites (strain 9750 at 65B2). *w+* embryos were selected and stable transgenic fly stocks established.

### Live fluorescent imaging of PRs

CO2-anesthetized flies were placed in a 35 mm cell culture dish half-filled with 1% agarose, covered with water at 4°C and observed using an upright 510 Zeiss confocal fluorescent microscope as described [20]. For the time-course study of age-dependent PR death in single flies, after visualization, each living fly was detached from the agarose, dried and transferred to a vial containing fly food medium.

### Resin-embedded tangential sections

Tangential sections of adult eyes were performed as described [45]. PR viability was determined by counting the number of intact rhabdomeres on retina tangential plastic sections. At least 200 ommatidia from three different animals were scored per experimental condition.

### Transmission electron microscopy

*Drosophila* eyes were dissected and fixed overnight at 4°C in 1.5% glutaraldehyde, 1% paraformaldehyde and 0.1M PIPES buffer (pH 7.4). After washing, eyes were post-fixed at room temperature in 1% OsO4, 0.1M PIPES (pH 7.4). They were then deshydrated with successive ethanol solutions followed by anhydrous propylen oxyde. Eyes were infiltrated with increasing concentrations of epoxy resin (EMbed 812 from EMS) in propylen oxyde for 1 day at room temperature and samples were mounted in pure resin into silicone embedding molds. Polymerization was performed at 60°C for 2 days. Ultrathin sections of 60 nm were stained with lead citrate and examined with a transmission electron microscope (Philips CM120) operating at 80 kV.

### Generation of anti-Fatp C11-7 antibody

Two rabbits were immunized with two peptides of Fatp, Peptide 1: YQTSKGRYELLTPQ at the C-terminus of the protein and Peptide 2: NNNSETEKNIPQAK in the middle of the protein. The serum from the two rabbits were pooled and affinity purified.

### Immunostainings

Horizontal eye cryosections were performed using a cryostat microtome (Microm HM505E) and deposited on superfrost Plus slides (Thermo). Third instar larval imaginal discs were dissected in 1X PBS. For whole-mount retina, we followed the protocol described in [46]. Briefly, *Drosophila* heads were bisected in the middle with a scalpel. Brain tissue was removed to expose retina underneath. Cryosections, imaginal discs and whole-mount retinae were fixed in 4% PFA for 15 min. After washing in PBS+Triton X-100 (0.3%), the following antibodies were used in PBS+Triton X-100 (0.1%)+Normal Goat Serum (5%, Sigma) overnight at 4°C: anti-fatp C11-7 (1/200), anti-ELAV (1/500, DSHB), anti-β Galactosidase (1/500, MP Cappell). After washing, samples were stained with the following appropriate secondary antibodies: anti-rabbit (Alexa 488 1/500, Invitrogen), anti-rat (alexa 633 1/500, Invitrogen). For whole-mount retina, phalloidin-rhodamine (1/200, Sigma) was also used to stain rhabdomeres. Samples were mounted in DAPI mounting media (Vectashield, AbCys). Fluorescent images were obtained using Zeiss 510 and 710 confocal microscopes.

### Electroretinogram

For ERG recordings, white-eyed flies were analyzed. Cold-anesthetized flies were immobilized in clay. A tungsten electrode (0.5–1 MΩ, Intracell) was inserted in the back of the head and a glass electrode filled with 3 M KCl (2–6 MΩ) was poked through the cornea. Flies were dark-adapted for 2 min before recording. An orange LED (591 nm, 2800 mcd, 40° beam, LY 5436-VBW-1, Osram, France) was placed at 1 cm from the head. The flash intensity reaching the eye was 650 µW/cm^2^, as measured with a PM100D power meter and S121C photodiode (Thorlabs, Maisons-Laffitte, France). Signals were filtered at 2 kHz and digitized at 10 kHz, using a MultiClamp 700A amplifier, a Digidata 1322A interface and pClamp-8 software (Molecular Devices, Sunnyvale, USA). Flash intensity and duration were controlled through pClamp and the Digidata analog output.

### Histological detection of β-galactosidase activity

Horizontal adult eye sections were performed using a cryostat microtome (Microm) and deposited on superfrost Plus slides (Thermo). Third instar laval imaginal discs, midgut and salivary gland were dissected in 1X PBS. Samples were fixed 5 min in PBS 0.25% gluteraldehyde. They were stained in a solution of 7.2 mM Na2HPO4, 2.8 mM NaH2PO4, 150 mM NaCl, 1 mM MgCl2, 3 mM K3[Fe(CN)6], 3 mM K4[Fe(CN)6], containing a 1/30 dilution of X-Gal (30 mg/ml in dimethyl formamide). After washing in PBS, samples were mounted in DAPI mounting media (Vectashield, AbCys).

### RT–PCR

mRNA was extracted from 20 retinas and 40 embryos using QIAshredder and RNeasy Mini kits (Qiagen). 100 ng of mRNA was used to synthesize cDNA using the Enhanced Avian RT First Strand Synthesis Kit (Sigma Aldrich) following manufacturer's instruction. Briefly, mRNA is incubated at 70°C for 10 min with dNTP (0.5 mM) and oligodT (3.5 µM) and then incubated at 50°C for 1 h with 1X buffer, reverse transcriptase enzyme (20U) and RNase inhibitor (20U). PCR was performed using GoTaq (Promega, 2U) in GreenGoTaq Buffer (Promega, 1X) with 200 µM dNTP and two pairs of primers (200 nM each, *fatp* forward: GGATTTTTGCTGTGCTCGTC, *fatp* reverse: ACCACATCGCCCTTTTTGTA, *rp49* forward: CGGATCGATATGCTAAGCTGT, *rp49* reverse: GCGCTTGTTCGATCCGTA). *rp49* amplification was used as an internal control. cDNA was first denatured for 5 min at 95°C and amplified during 35 cycles: 95°C for 30 s, 59°C for 30 s, 72°C for 42 s; followed by an incubation at 72°C for 7 min. Amplified cDNA was segregated in 1.5% agarose gel.

### Western blot

10 *Drosophila* adult heads were homogenized in 30 µL Laemmli buffer (10% glycerol, pH 6.8 0.5M Tris, 10% SDS, 1% bromophenol blue, 1% β-mercaptoethanol, 100 mM DTT) and centrifuged for 30 min at 12,000 g. Supernatant was boiled for 5 min and 10 µL was loaded onto a 12% acrylamide gel (Biorad) and transferred onto nitrocellulose membranes (Whatman). Anti-Fatp C11-7 (1/200), anti-Rh1 (1/1000, 4C5, DSHB) and anti-Tubulin (1/1000, Sigma) antibodies were incubated overnight at 4°C and appropriate HRP-coupled secondary anti-mouse and anti-rabbit antibodies (1∶10 000, Biorad) were then incubated for 2 h at RT. Chemiluminescent detection was carried out using a ECL kit (GE Healthcare Life Sciences). Protein band quantification was carried out using ImageJ software.

For blue light-induced degradation of Rh1, white-eyed heads were exposed to 10 mW blue light for 6 h and homogenized in Tris-buffered saline (20 mM Tris (pH 7.5), 150 mM NaCl) containing 0.5% Triton X-100 and protease inhibitors. After centrifugation, the supernatant was mixed with an equal volume of 2X Laemmli buffer and loaded onto a 12% acrylamide gel as described above.

## Supporting Information

Figure S1 Recessive lethal *fatp^k10307^* mutation is protein null. (A) The *fatp^k10307^* mutation consists of the insertion of a P{lacW} element (10,7 kb) in the 5′ end of the open reading frames. We assumed that it disrupts *fatp* expression. In addition, the P{lacW} element carries a lacZ sequence which captures *fatp* expression profile. We first checked that *fatp^k10307^*-associated lethality is localized in the *fatp* locus. We determined that *fatp^k10307^* was trans-heterozygous lethal over *Df(2L)Exel7048* and *Df(2L)BSC210*, two deficiencies (101.5 and 199.6 kb respectively) covering the *fatp* locus and was not lethal over *Df(2L)BSC342*, a nearby deficiency that does not cover *fatp* locus. (B–E) Photographes of first and second wild-type *Canton-S* (*Cs*) and *fatp^k10307/k10307^* mutant larvae (scale bar = 0.5 mm). First instar larvae (L1, B,C) were observed 42–47 h after egg laying and second instar larvae (L2, D, E) 67–71 h after egg laying. Whereas first instar homozygous mutant larvae are similar to willd type larvae in terms of size and mobility, second instar mutant larvae are dying as weakly mobile and small larvae. Their mouth hook is similar to second instar larvae (F, scale bar = 15 µm). This result indicates that *fatp^k10307^* mutation is lethal in L2 instar. (G) Semi-quantitative duplex RT-PCR of *fatp* and *rp49* mRNA in wild-type and *fatp^k10307/k10307^* mutant retina and first instar larvae extract. In *fatp^k10307/k10307^*, no amplification of *fatp* mRNA is detected suggesting that *fatp^k10307^* is a null mutation. *rp49* is used as an internal control. (H) Western blot analysis of Fatp and Tubulin in wild-type and *fatp^k10307/k10307^* mutant first instar larvae extract. In the mutant condition, Fatp is not detected. Tubulin is used as an internal control. Thus *fatp^k10307^* mutation is protein null.
(TIF)

Figure S2 Progressive mis-localization of PR nuclei in *fatp* mutant retina. (A–G) Nucleus visualization of 21 day-old (A, B), 28 day-old (C, D), 35 day-old (E, F), control (A, C, E) and homozygous whole-eye clone (B, D, F). Nuclei are stained with DAPI on cryosections (scale bar = 100 µm). Localization of nuclei between the proximal and distal part of the retina (red circles) is abnormal and corresponds to nuclei of dying PRs. (G) Quantification of the nuclei of dying PRs. Mutant retina exhibits significantly higher nuclei of dying PRs than control retina (test-t, n> = 6). Difference between control and mutant retina is more pronounced with age.
(TIF)

Figure S3 *fatp* expression by detection of lacZ activity in adult eye and third instar larva organs. (A–D) Immunostaining of lacZ and ELAV in a *fatp^k10307/+^* head cryosection (scale bar = 50 µm). On a close-up view of the retina distal part (B, C and D), the lacZ staining colocalized with the ELAV staining (arrowhead). It is also located distally at the level of IOCs (arrow). (E–L) Detection of lacZ activity in *Cs* (E–H) and *fatp^k10307/+^* (I–L) organs. *fatp^k10307^* is an enhancer trap in which *lacZ* is expressed according to the expression profil of *fatp*. (E, I) Horizontal cryosection of heads (scale bar = 100 µm). *fatp* is expressed specifically in the retina (*) at the level of PRs and IOCs (arrow) and in cells around the lamina (#) and the medulla (¤) (arrowhead). (F, J) third instar larva imaginal discs. No lacZ activity is detected. (G, K) lacZ activity is detected in the midgut (arrow) and in the posterior part of the proventriculus (arrowhead). (H, L) lacZ activity is expressed in the anterior part of the salivary gland (arrowheads).
(TIF)

Figure S4 Retinal pathology in *rh1^G69D^* flies exposed to constant illumination. White-eyed control and *rh1^G69D/+^* flies (A, C, E, G and B, D, F, H) were reared under constant illumination for 15 and 21 days (A, B, E, F and C, D, G, H). Their retinas were analyzed using plastic section (A–D) and electron microscopy (E–H). In the *rh1^G69D/+^* flies, the rhabdomere size of the outer PRs is smaller than in the control (arrow, compare with rhabdomere of inner PR (*) as an internal control). At 21-day-old, outer *rh1^G69D/+^* PRs are degenerating (arrows).
(TIF)

Figure S5 *Rh1^I17^* rescues PR viability in the *fatp* mutant. (A, B) Analysis of the survival of whole eye *fatp^k10307^* clone and *fatp^k10307^ rh1^I17/+^* double mutant PRs using resin-embedded tangential sections in 28-day-old flies (scale bar = 10 µm). (C) Quantification of PR cell death. In the double mutant, PR loss was significantly reduced in comparison with the *fatp* mutant (t-test, n = 4).
(TIF)

Figure S6 Pathological consequences of vitamin A deprivation on PRs. (A, B) Analysis of control and vitaminA-deprived 23-day-old flies using plastic section. The size of the rhabdomeres is clearly reduced in vitaminA-deprived flies vs control flies. Some photoreceptors are missing. (C, D) Analysis of the same flies using electron microscopy. In vitaminA-deprived flies, the size of rhabdomeres is reduced or replaced by subrhabdomeric membrane processes.
(TIF)

Figure S7 Overexpression of Rh1 in the eye is toxic. (A, B) Images of the eye of 3-day-old flies overexpressing lacZ as a control (A) and *rh1* (B). Flies that overexpressed *rh1* had a rough eye phenotype (B). (C, D) Electron microscopy analysis of *wt* retina overexpressing lacZ (C) as a control and *rh1* (D), under the *rh1* promoter. In *rh1* overexpressing ommatidia, PRs were degenerating. The degeneration started with the appearance of vacuoles in the rhabdomere (*), which accumulated until the rhabdomere was totally disorganized (arrows). The arrowhead corresponds to an experimental artifact.
(TIF)

Figure S8 Loss of *fatp* reduces light-induced Rh1 degradation. (A, B) Analysis of *rh1* promoter activity using the *rh1-lacZ* reporter line in whole-eye control and *fatp^k10307^* mutant retinas. (A) Western blot analysis of LacZ levels in control and *fatp^k10307^* heads. Tubulin was used as a loading control. (B) quantification of protein levels. The expression of LacZ was similar in control and *fatp* mutant retinas. (C) Western blot analysis of Rh1 in unboiled head extracts from control and *fatp^k10307/k10307^* flies exposed to blue light. Heads were either kept in the dark (light −) or exposed to blue light for 6 hours (light +). Tubulin was used as a loading control. (D) Quantification of protein levels. Whereas Rh1 levels are twofold decreased after light illumination in control heads, Rh1 levels are not significantly decreased in *fatp* mutant heads.
(TIF)

## References

[1] Sohocki MM, Daiger SP, Bowne SJ, Rodriquez JA, Northrup H (2001). Prevalence of mutations causing retinitis pigmentosa and other inherited retinopathies.. Hum Mutat.

[2] Lee ES, Flannery JG (2007). Transport of truncated rhodopsin and its effects on rod function and degeneration.. Invest Ophthalmol Vis Sci.

[3] Mollereau B, Domingos PM (2005). Photoreceptor differentiation in Drosophila: from immature neurons to functional photoreceptors.. Dev Dyn.

[4] Wang T, Montell C (2007). Phototransduction and retinal degeneration in Drosophila.. Pflugers Arch.

[5] Shieh BH (2011). Molecular genetics of retinal degeneration: A Drosophila perspective.. Fly (Austin).

[6] Galy A, Roux MJ, Sahel JA, Leveillard T, Giangrande A (2005). Rhodopsin maturation defects induce photoreceptor death by apoptosis: a fly model for RhodopsinPro23His human retinitis pigmentosa.. Hum Mol Genet.

[7] Gambis A, Dourlen P, Steller H, Mollereau B (2011). Two-color in vivo imaging of photoreceptor apoptosis and development in Drosophila.. Dev Biol.

[8] Watkins PA, Maiguel D, Jia Z, Pevsner J (2007). Evidence for 26 distinct acyl-coenzyme A synthetase genes in the human genome.. J Lipid Res.

[9] Watkins PA (2008). Very-long-chain acyl-CoA synthetases.. J Biol Chem.

[10] Gimeno RE (2007). Fatty acid transport proteins.. Curr Opin Lipidol.

[11] Schaffer JE, Lodish HF (1994). Expression cloning and characterization of a novel adipocyte long chain fatty acid transport protein.. Cell.

[12] Guignard TJ, Jin M, Pequignot MO, Li S, Chassigneux Y (2010). FATP1 inhibits 11-cis retinol formation via interaction with the visual cycle retinoid isomerase RPE65 and lecithin:retinol acyltransferase.. J Biol Chem.

[13] Herrmann T, Buchkremer F, Gosch I, Hall AM, Bernlohr DA (2001). Mouse fatty acid transport protein 4 (FATP4): characterization of the gene and functional assessment as a very long chain acyl-CoA synthetase.. Gene.

[14] Stahl A (2004). A current review of fatty acid transport proteins (SLC27).. Pflugers Arch.

[15] Herrmann T, van der Hoeven F, Grone HJ, Stewart AF, Langbein L (2003). Mice with targeted disruption of the fatty acid transport protein 4 (Fatp 4, Slc27a4) gene show features of lethal restrictive dermopathy.. J Cell Biol.

[16] Moulson CL, Martin DR, Lugus JJ, Schaffer JE, Lind AC (2003). Cloning of wrinkle-free, a previously uncharacterized mouse mutation, reveals crucial roles for fatty acid transport protein 4 in skin and hair development.. Proc Natl Acad Sci U S A.

[17] Klar J, Schweiger M, Zimmerman R, Zechner R, Li H (2009). Mutations in the fatty acid transport protein 4 gene cause the ichthyosis prematurity syndrome.. Am J Hum Genet.

[18] Golic KG (1991). Site-specific recombination between homologous chromosomes in Drosophila.. Science.

[19] Mollereau B, Wernet MF, Beaufils P, Killian D, Pichaud F (2000). A green fluorescent protein enhancer trap screen in Drosophila photoreceptor cells.. Mech Dev.

[20] Pichaud F, Desplan C (2001). A new visualization approach for identifying mutations that affect differentiation and organization of the Drosophila ommatidia.. Development.

[21] Brand AH, Perrimon N (1993). Targeted gene expression as a means of altering cell fates and generating dominant phenotypes.. Development.

[22] Stowers RS, Schwarz TL (1999). A genetic method for generating Drosophila eyes composed exclusively of mitotic clones of a single genotype.. Genetics.

[23] Kurada P, O'Tousa JE (1995). Retinal degeneration caused by dominant rhodopsin mutations in Drosophila.. Neuron.

[24] Colley NJ, Cassill JA, Baker EK, Zuker CS (1995). Defective intracellular transport is the molecular basis of rhodopsin-dependent dominant retinal degeneration.. Proc Natl Acad Sci U S A.

[25] Harris WA, Stark WS (1977). Hereditary retinal degeneration in Drosophila melanogaster. A mutant defect associated with the phototransduction process.. J Gen Physiol.

[26] Alloway PG, Howard L, Dolph PJ (2000). The formation of stable rhodopsin-arrestin complexes induces apoptosis and photoreceptor cell degeneration.. Neuron.

[27] Johnson K, Grawe F, Grzeschik N, Knust E (2002). Drosophila crumbs is required to inhibit light-induced photoreceptor degeneration.. Curr Biol.

[28] Chinchore Y, Mitra A, Dolph PJ (2009). Accumulation of rhodopsin in late endosomes triggers photoreceptor cell degeneration.. PLoS Genet.

[29] Satoh AK, Ready DF (2005). Arrestin1 mediates light-dependent rhodopsin endocytosis and cell survival.. Curr Biol.

[30] Kiselev A, Socolich M, Vinos J, Hardy RW, Zuker CS (2000). A molecular pathway for light-dependent photoreceptor apoptosis in Drosophila.. Neuron.

[31] Xu H, Lee SJ, Suzuki E, Dugan KD, Stoddard A (2004). A lysosomal tetraspanin associated with retinal degeneration identified via a genome-wide screen.. Embo J.

[32] Acharya U, Patel S, Koundakjian E, Nagashima K, Han X (2003). Modulating sphingolipid biosynthetic pathway rescues photoreceptor degeneration.. Science.

[33] Yonamine I, Bamba T, Nirala NK, Jesmin N, Kosakowska-Cholody T (2011). Sphingosine kinases and their metabolites modulate endolysosomal trafficking in photoreceptors.. J Cell Biol.

[34] Acharya U, Mowen MB, Nagashima K, Acharya JK (2004). Ceramidase expression facilitates membrane turnover and endocytosis of rhodopsin in photoreceptors.. Proc Natl Acad Sci U S A.

[35] Wang X, Wang T, Jiao Y, von Lintig J, Montell C (2010). Requirement for an enzymatic visual cycle in Drosophila.. Curr Biol.

[36] Wang X, Wang T, Ni JD, von Lintig J, Montell C (2012). The Drosophila visual cycle and de novo chromophore synthesis depends on rdhB.. J Neurosci.

[37] Montell C (2012). Drosophila visual transduction.. Trends Neurosci.

[38] Lee SJ, Xu H, Montell C (2004). Rhodopsin kinase activity modulates the amplitude of the visual response in Drosophila.. Proc Natl Acad Sci U S A.

[39] Hardie RC, Martin F, Chyb S, Raghu P (2003). Rescue of light responses in the Drosophila “null” phospholipase C mutant, norpAP24, by the diacylglycerol kinase mutant, rdgA, and by metabolic inhibition.. J Biol Chem.

[40] Chyb S, Raghu P, Hardie RC (1999). Polyunsaturated fatty acids activate the Drosophila light-sensitive channels TRP and TRPL.. Nature.

[41] Leung HT, Tseng-Crank J, Kim E, Mahapatra C, Shino S (2008). DAG lipase activity is necessary for TRP channel regulation in Drosophila photoreceptors.. Neuron.

[42] Mendes CS, Levet C, Chatelain G, Dourlen P, Fouillet A (2009). ER stress protects from retinal degeneration.. EMBO J.

[43] Lee YS, Carthew RW (2003). Making a better RNAi vector for Drosophila: use of intron spacers.. Methods.

[44] Fish MP, Groth AC, Calos MP, Nusse R (2007). Creating transgenic Drosophila by microinjecting the site-specific phiC31 integrase mRNA and a transgene-containing donor plasmid.. Nat Protoc.

[45] Domingos PM, Mlodzik M, Mendes CS, Brown S, Steller H (2004). Spalt transcription factors are required for R3/R4 specification and establishment of planar cell polarity in the Drosophila eye.. Development.

[46] Domingos PM, Brown S, Barrio R, Ratnakumar K, Frankfort BJ (2004). Regulation of R7 and R8 differentiation by the spalt genes.. Dev Biol.

