# Supplementary figures and images for: Drosophila Fatty Acid Transport Protein Regulates Rhodopsin-1 Metabolism and Is Required for Photoreceptor Neuron Survival
Source: PLoS Genet. 2012 Jul 26;8(7):e1002833. doi: 10.1371/journal.pgen.1002833 (PMC3405995; doi:10.1371/journal.pgen.1002833)

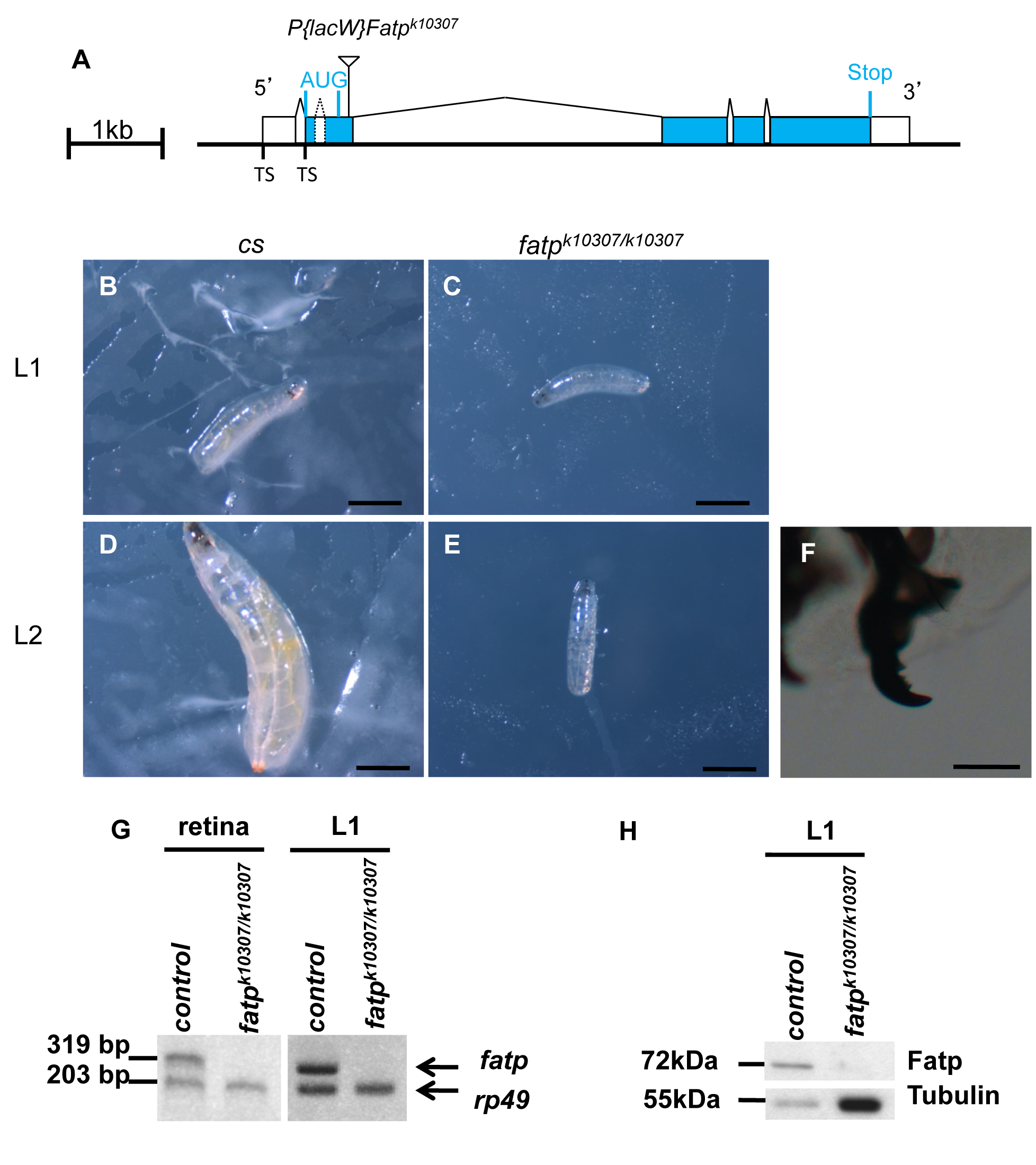

Supplement: Figure S1 — Recessive lethal fatpk10307 mutation is protein null. (A) The fatpk10307 mutation consists of the insertion of a P{lacW} element (10,7 kb) in the 5′ end of the open reading frames. We assumed that it disrupts fatp expression. In addition, the P{lacW} element carries a lacZ sequence which captures fatp expression profile. We first checked that fatpk10307-associated lethality is localized in the fatp locus. We determined that fatpk10307 was trans-heterozygous lethal over Df(2L)Exel7048 and Df(2L)BSC210, two deficiencies (101.5 and 199.6 kb respectively) covering the fatp locus and was not lethal over Df(2L)BSC342, a nearby deficiency that does not cover fatp locus. (B–E) Photographes of first and second wild-type Canton-S (Cs) and fatpk10307/k10307 mutant larvae (scale bar = 0.5 mm). First instar larvae (L1, B,C) were observed 42–47 h after egg laying and second instar larvae (L2, D, E) 67–71 h after egg laying. Whereas first instar homozygous mutant larvae are similar to willd type larvae in terms of size and mobility, second instar mutant larvae are dying as weakly mobile and small larvae. Their mouth hook is similar to second instar larvae (F, scale bar = 15 µm). This result indicates that fatpk10307 mutation is lethal in L2 instar. (G) Semi-quantitative duplex RT-PCR of fatp and rp49 mRNA in wild-type and fatpk10307/k10307 mutant retina and first instar larvae extract. In fatpk10307/k10307, no amplification of fatp mRNA is detected suggesting that fatpk10307 is a null mutation. rp49 is used as an internal control. (H) Western blot analysis of Fatp and Tubulin in wild-type and fatpk10307/k10307 mutant first instar larvae extract. In the mutant condition, Fatp is not detected. Tubulin is used as an internal control. Thus fatpk10307 mutation is protein null. (TIF) [file pgen.1002833.s001.tif]

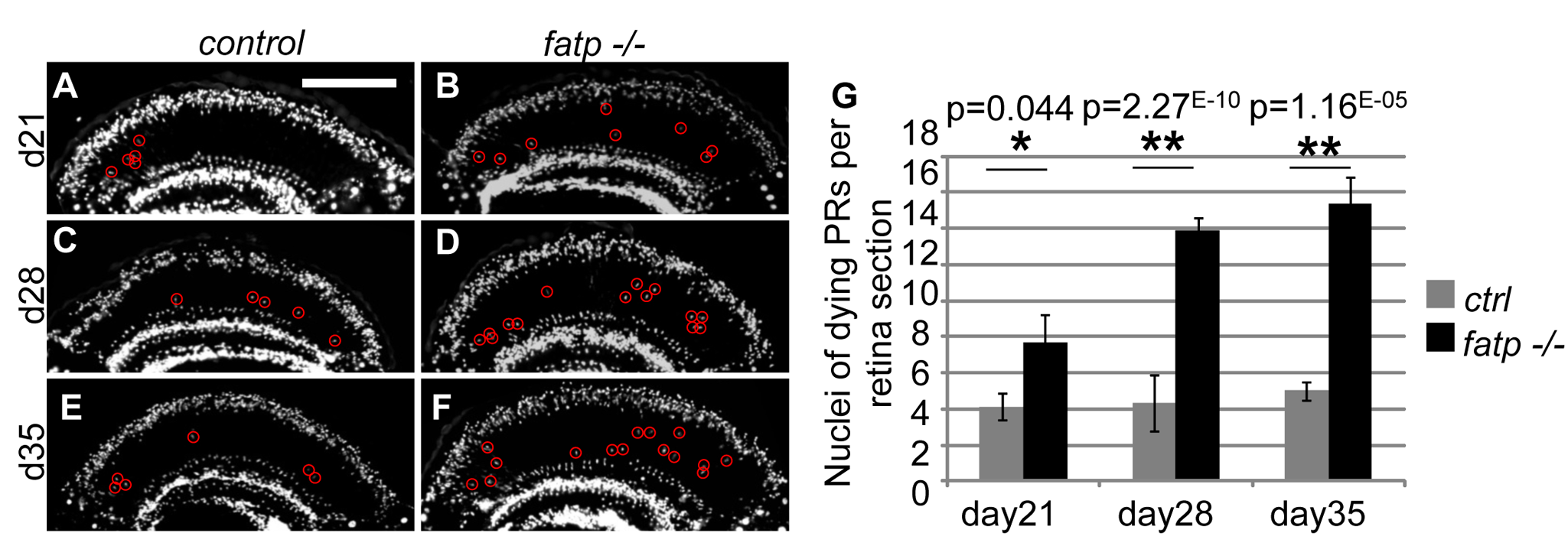

Supplement: Figure S2 — Progressive mis-localization of PR nuclei in fatp mutant retina. (A–G) Nucleus visualization of 21 day-old (A, B), 28 day-old (C, D), 35 day-old (E, F), control (A, C, E) and homozygous whole-eye clone (B, D, F). Nuclei are stained with DAPI on cryosections (scale bar = 100 µm). Localization of nuclei between the proximal and distal part of the retina (red circles) is abnormal and corresponds to nuclei of dying PRs. (G) Quantification of the nuclei of dying PRs. Mutant retina exhibits significantly higher nuclei of dying PRs than control retina (test-t, n> = 6). Difference between control and mutant retina is more pronounced with age. (TIF) [file pgen.1002833.s002.tif]

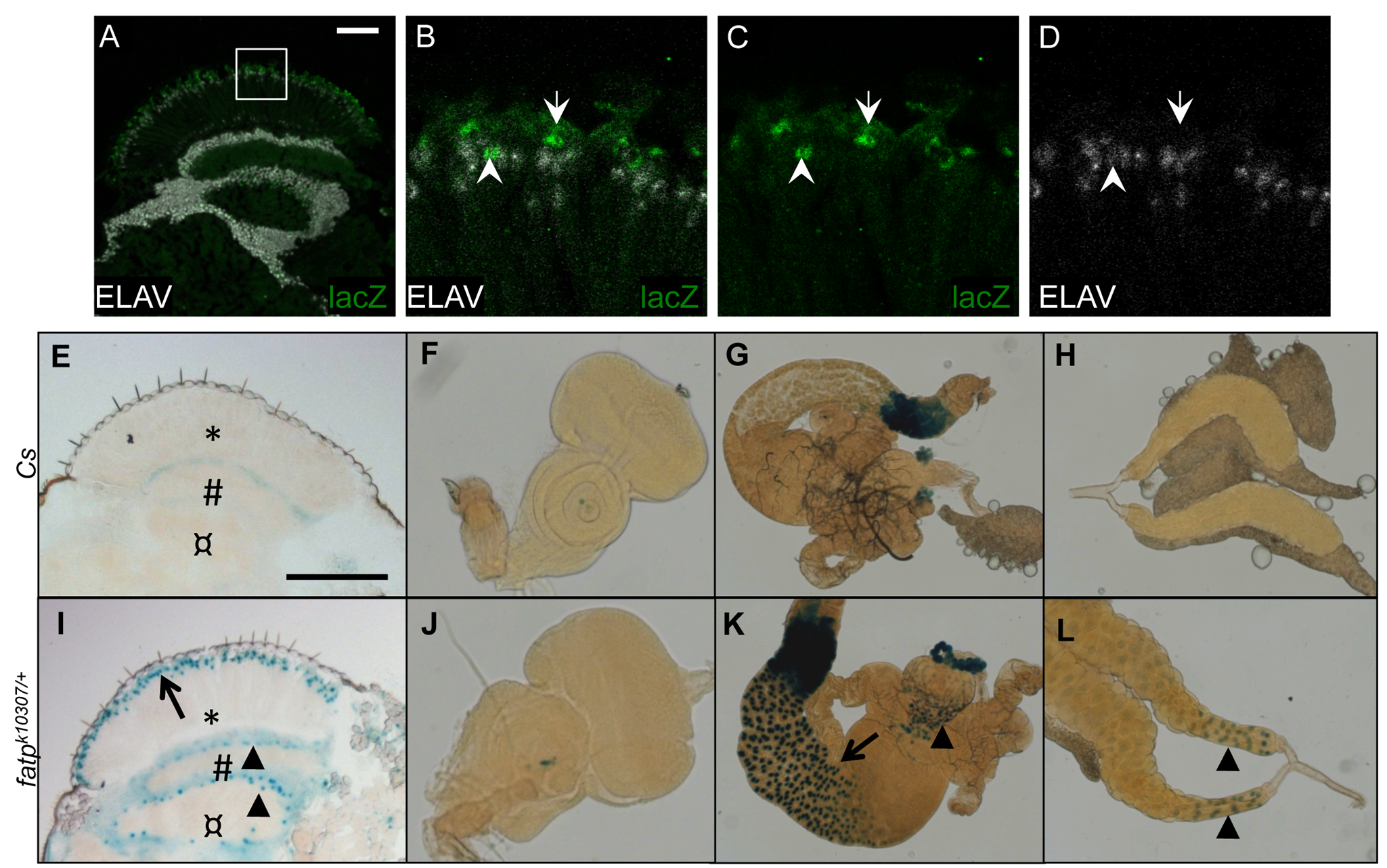

Supplement: Figure S3 — fatp expression by detection of lacZ activity in adult eye and third instar larva organs. (A–D) Immunostaining of lacZ and ELAV in a fatpk10307/+ head cryosection (scale bar = 50 µm). On a close-up view of the retina distal part (B, C and D), the lacZ staining colocalized with the ELAV staining (arrowhead). It is also located distally at the level of IOCs (arrow). (E–L) Detection of lacZ activity in Cs (E–H) and fatpk10307/+ (I–L) organs. fatpk10307 is an enhancer trap in which lacZ is expressed according to the expression profil of fatp. (E, I) Horizontal cryosection of heads (scale bar = 100 µm). fatp is expressed specifically in the retina (*) at the level of PRs and IOCs (arrow) and in cells around the lamina (#) and the medulla (¤) (arrowhead). (F, J) third instar larva imaginal discs. No lacZ activity is detected. (G, K) lacZ activity is detected in the midgut (arrow) and in the posterior part of the proventriculus (arrowhead). (H, L) lacZ activity is expressed in the anterior part of the salivary gland (arrowheads). (TIF) [file pgen.1002833.s003.tif]

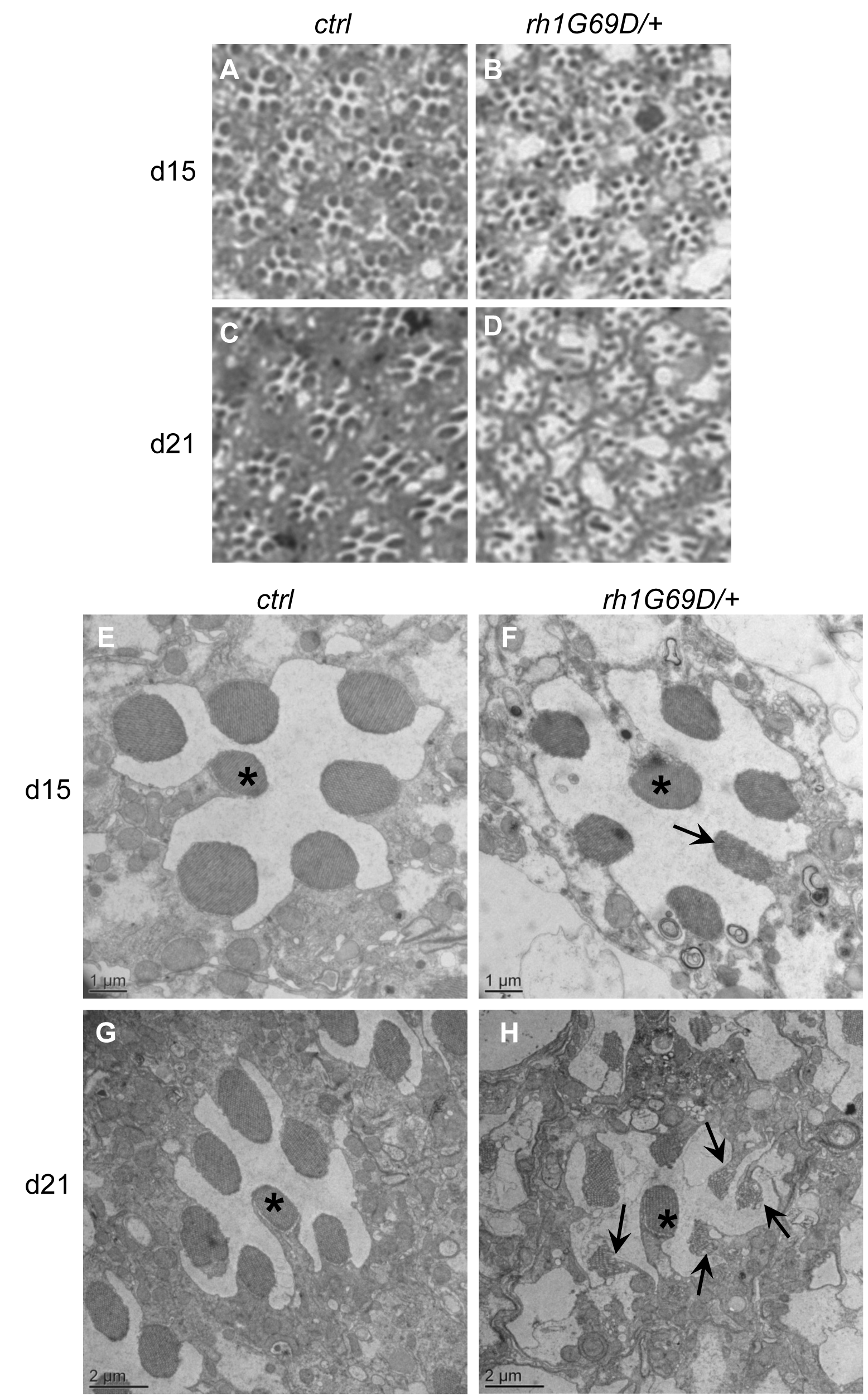

Supplement: Figure S4 — Retinal pathology in rh1G69D flies exposed to constant illumination. White-eyed control and rh1G69D/+ flies (A, C, E, G and B, D, F, H) were reared under constant illumination for 15 and 21 days (A, B, E, F and C, D, G, H). Their retinas were analyzed using plastic section (A–D) and electron microscopy (E–H). In the rh1G69D/+ flies, the rhabdomere size of the outer PRs is smaller than in the control (arrow, compare with rhabdomere of inner PR (*) as an internal control). At 21-day-old, outer rh1G69D/+ PRs are degenerating (arrows). (TIF) [file pgen.1002833.s004.tif]

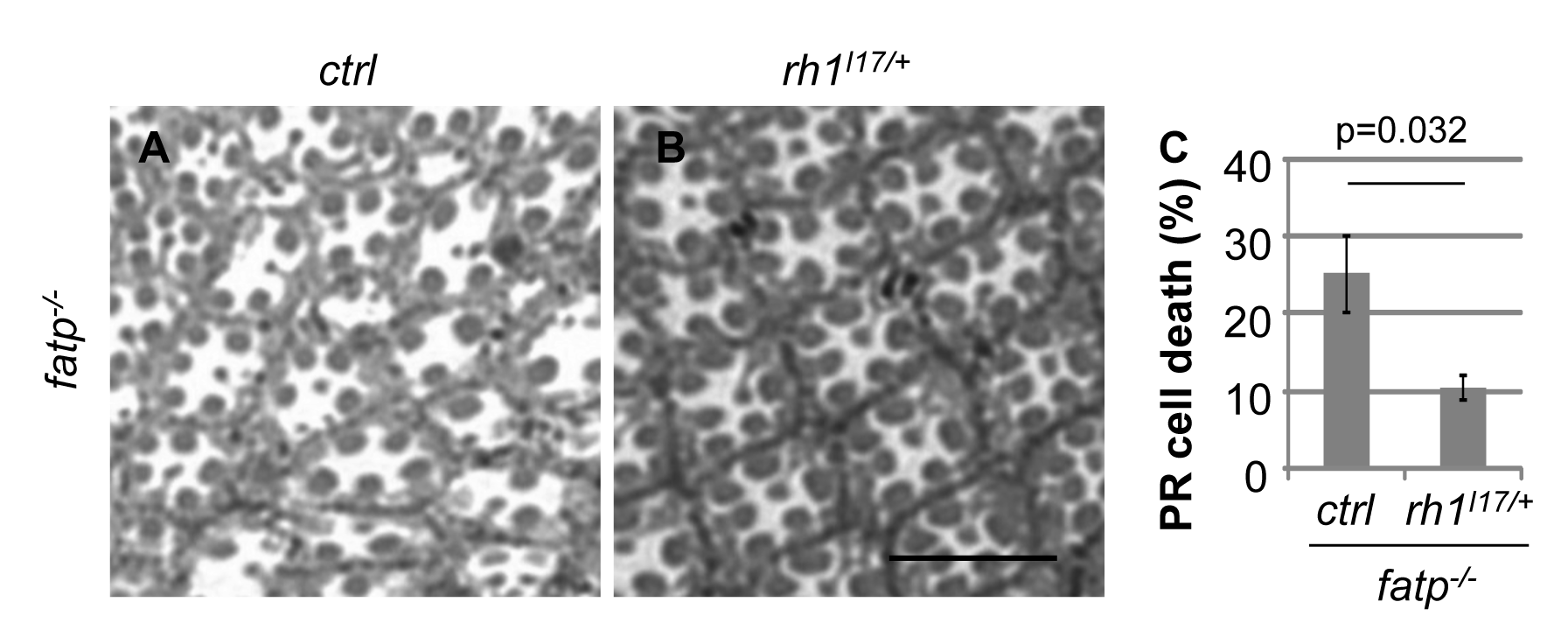

Supplement: Figure S5 — Rh1I17 rescues PR viability in the fatp mutant. (A, B) Analysis of the survival of whole eye fatpk10307 clone and fatpk10307 rh1I17/+ double mutant PRs using resin-embedded tangential sections in 28-day-old flies (scale bar = 10 µm). (C) Quantification of PR cell death. In the double mutant, PR loss was significantly reduced in comparison with the fatp mutant (t-test, n = 4). (TIF) [file pgen.1002833.s005.tif]

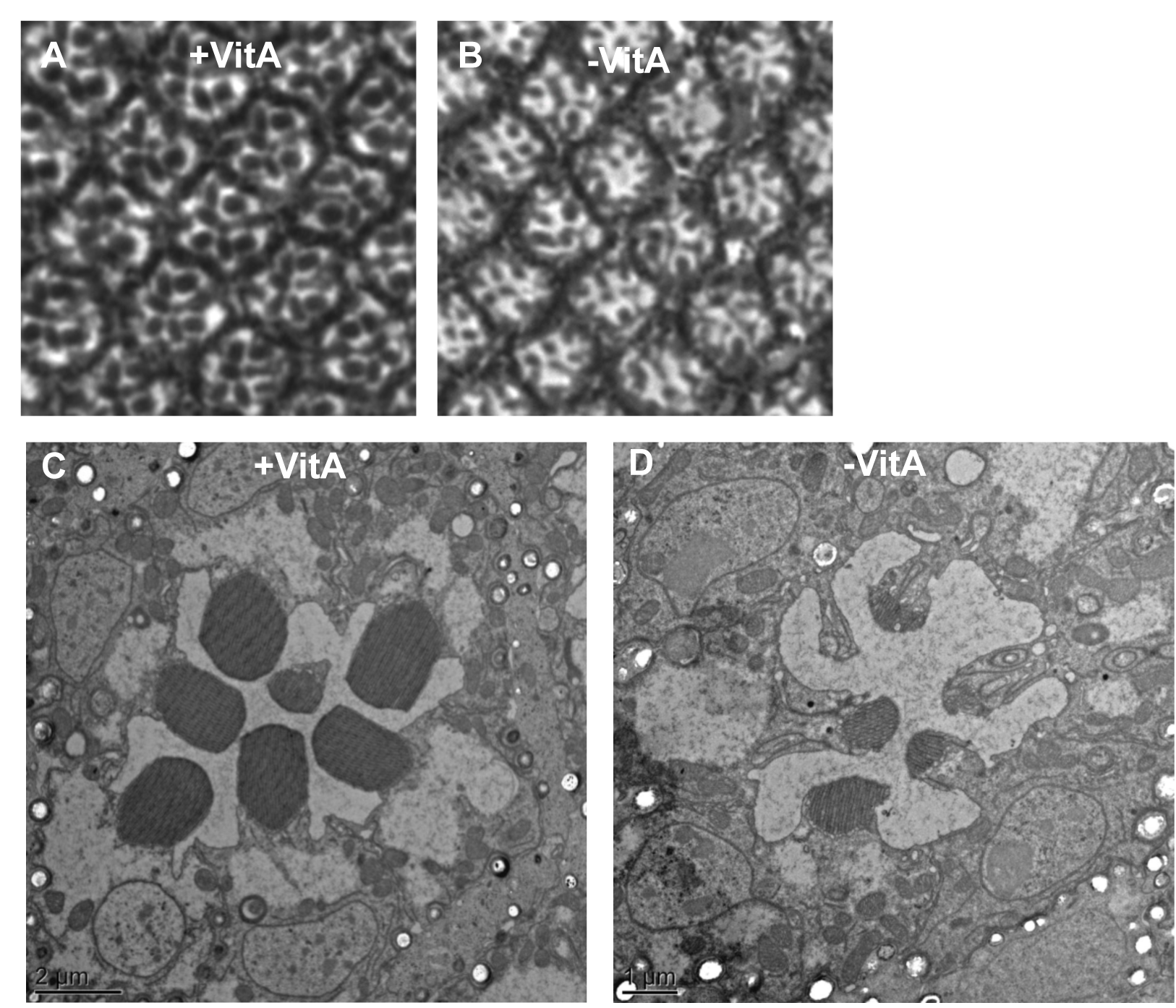

Supplement: Figure S6 — Pathological consequences of vitamin A deprivation on PRs. (A, B) Analysis of control and vitaminA-deprived 23-day-old flies using plastic section. The size of the rhabdomeres is clearly reduced in vitaminA-deprived flies vs control flies. Some photoreceptors are missing. (C, D) Analysis of the same flies using electron microscopy. In vitaminA-deprived flies, the size of rhabdomeres is reduced or replaced by subrhabdomeric membrane processes. (TIF) [file pgen.1002833.s006.tif]

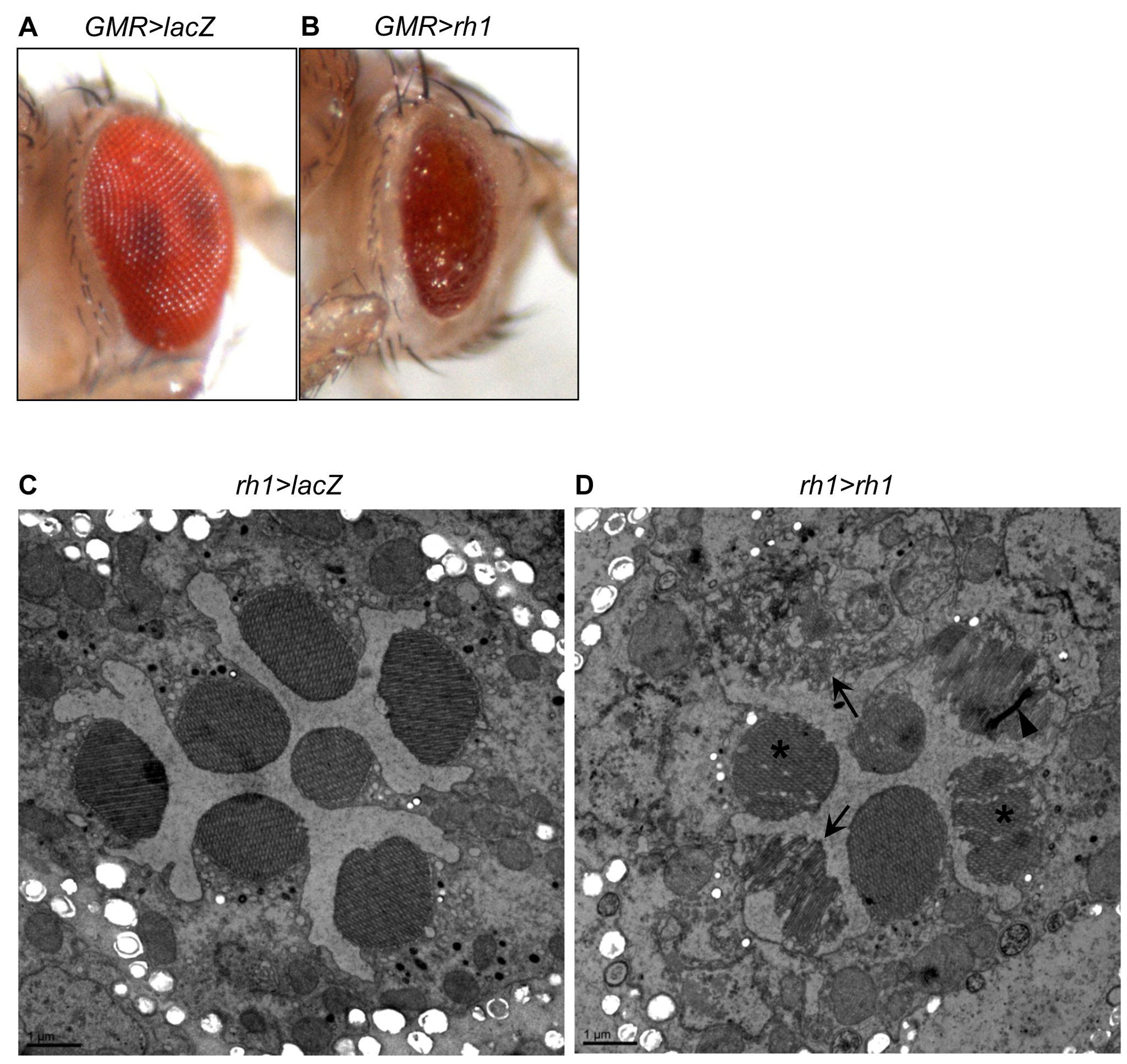

Supplement: Figure S7 — Overexpression of Rh1 in the eye is toxic. (A, B) Images of the eye of 3-day-old flies overexpressing lacZ as a control (A) and rh1 (B). Flies that overexpressed rh1 had a rough eye phenotype (B). (C, D) Electron microscopy analysis of wt retina overexpressing lacZ (C) as a control and rh1 (D), under the rh1 promoter. In rh1 overexpressing ommatidia, PRs were degenerating. The degeneration started with the appearance of vacuoles in the rhabdomere (*), which accumulated until the rhabdomere was totally disorganized (arrows). The arrowhead corresponds to an experimental artifact. (TIF) [file pgen.1002833.s007.tif]

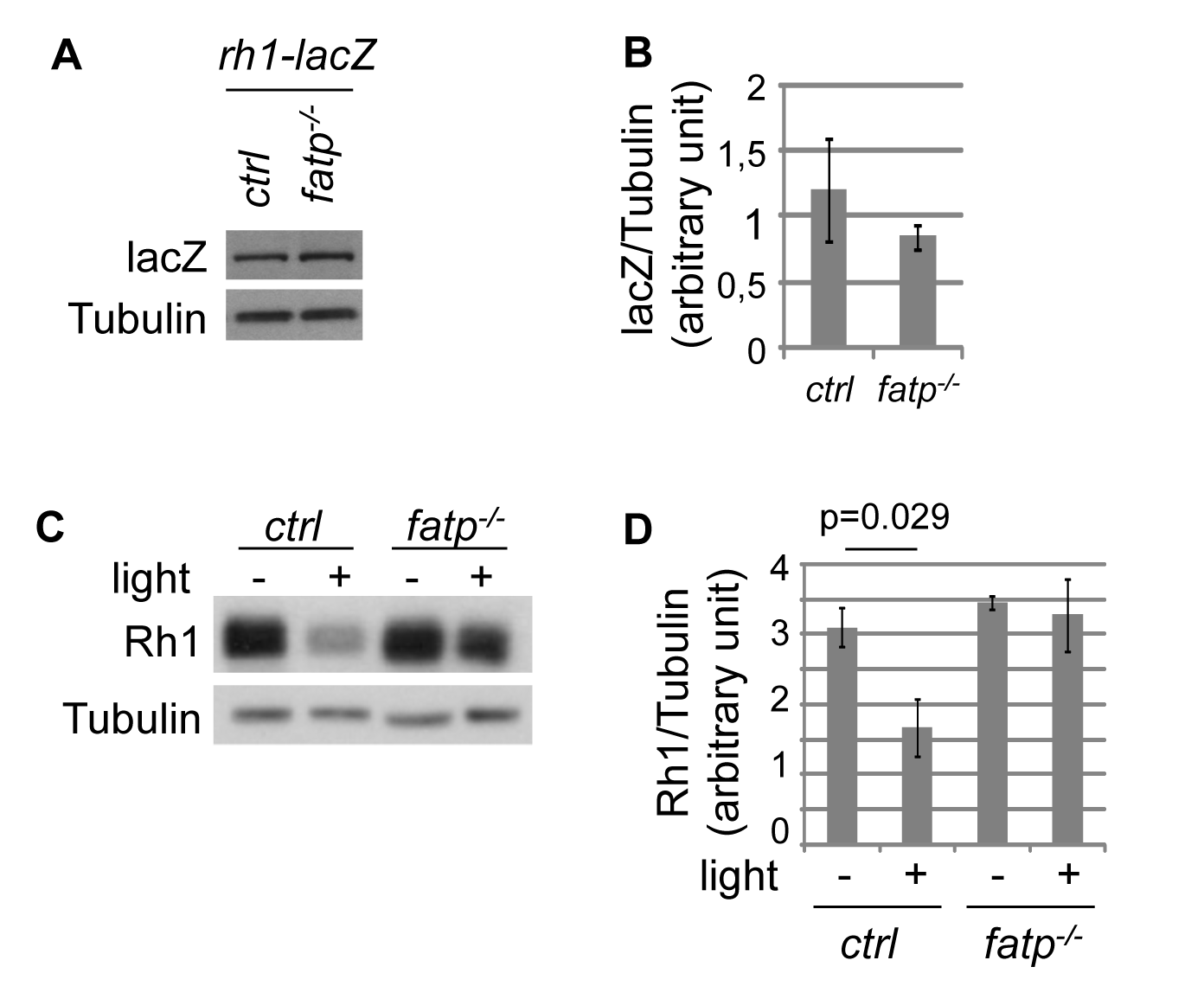

Supplement: Figure S8 — Loss of fatp reduces light-induced Rh1 degradation. (A, B) Analysis of rh1 promoter activity using the rh1-lacZ reporter line in whole-eye control and fatpk10307 mutant retinas. (A) Western blot analysis of LacZ levels in control and fatpk10307 heads. Tubulin was used as a loading control. (B) quantification of protein levels. The expression of LacZ was similar in control and fatp mutant retinas. (C) Western blot analysis of Rh1 in unboiled head extracts from control and fatpk10307/k10307 flies exposed to blue light. Heads were either kept in the dark (light −) or exposed to blue light for 6 hours (light +). Tubulin was used as a loading control. (D) Quantification of protein levels. Whereas Rh1 levels are twofold decreased after light illumination in control heads, Rh1 levels are not significantly decreased in fatp mutant heads. (TIF) [file pgen.1002833.s008.tif]
